# Immunological role and clinical prognostic significance of P2RY6 in lung adenocarcinoma: a multi-omics studies and single-cell sequencing analysis

**DOI:** 10.1186/s12957-023-03216-1

**Published:** 2023-10-26

**Authors:** Hong Wu, Xuhui Dong

**Affiliations:** Department of Pneumology, Yiwu Central Hospital, Zhejiang, China

**Keywords:** P2RY6, Lung adenocarcinoma, Immunotherapy, Pan-cancer, Clinical prognostic

## Abstract

**Background:**

There is increasing evidence that recombinant human P2Y purinoceptor 6 (P2RY6) may be involved in inflammatory responses. However, the role of P2RY6 in lung adenocarcinoma (LUAD) remains unknown.

**Methods:**

We used transcriptomic, genomic, single-cell transcriptomic, and methylation sequencing data from The Cancer Genome Atlas database to analyze the aberrant status and prognostic value of P2RY6 in a variety of tumors. The LUAD single-cell sequencing dataset was used to explore the effect of P2RY6 on the tumor microenvironment. Cell-type identification by estimating relative subsets of RNA transcripts (CIBERSORT) was used to quantify immune cells in the tumor microenvironment. We also analyzed the correlation of P2RY6 with immune checkpoints and immune regulation-related genes. The correlation of between tumor mutation burden (TMB), microsatellite instability (MSI), and P2RY6 expression was also analyzed simultaneously. Tissue microarray and immunohistochemistry were employed to assess the expression of P2RY6 in internal tumor samples.

**Results:**

Our findings indicate that P2RY6 exhibits significantly higher expression levels in various cancer tissues, particularly in LUAD. High expression of P2RY6 was closely associated with a poor prognosis for patients, and it plays a role in regulating immune-related pathways, such as cytokine-cytokine receptor interaction. Notably, P2RY6 expression is closely linked to the abundance of CD8 + T cells. Furthermore, we have developed a P2RY6-related inflammation prediction model that demonstrates promising results in predicting the prognosis of LUAD patients, with an AUC (area under the curve) value of 0.83. This performance is significantly better than the traditional TNM staging system. Through single-cell transcriptome sequencing analysis, we observed that high P2RY6 expression is associated with increased intercellular communication. Additionally, pathway enrichment analysis revealed that P2RY6 influences antigen presentation and processing pathways within the LUAD microenvironment.

**Conclusions:**

This study suggests that P2RY6 would be a new target for immunotherapy in LUAD.

**Supplementary Information:**

The online version contains supplementary material available at 10.1186/s12957-023-03216-1.

## Introduction

Lung cancer is currently the most prevalent form of malignancy worldwide, with the highest incidence and mortality rates [1]. It can be classified into two subtypes: small cell lung cancer (SCLC) and non-small cell lung cancer (NSCLC). NSCLC accounts for 85% of all cases [2]. Among NSCLC, lung adenocarcinoma (LUAD) is the most prevalent histological type of primary lung tumor [3]. Current treatment options for lung cancer include targeted therapy and immunotherapy, in combination with traditional surgery, radiotherapy, and chemotherapy. These advancements have significantly improved the survival rate of patients [4, 5]. However, due to diagnostic limitations, many LUAD patients are diagnosed at advanced stages of the disease, often accompanied by metastasis, resulting in a poor prognosis [6]. Despite extensive research on the pathogenesis of LUAD and the development of new treatment approaches, it unfortunately remains one of the most aggressive and deadly types of tumors, with a 5-year survival rate of approximately 23% [7]. In recent years, immune checkpoint inhibitors have demonstrated strong efficacy in many types of cancer [8–10]. The tumor microenvironment of the lung is known to be infiltrated by a diverse range of immune cells [11, 12], including natural killer cells, B cells, macrophages, dendritic cells, and T lymphocytes. As such, it has been suggested that LUAD may be a “hot” tumor type, and could be particularly sensitive to immunotherapy [13, 14]. Exploring new immunotherapy targets of LUAD and exploring the immune escape mechanism of LUAD is of great significance for improving the prognosis of LUAD patients.

P2RY6, also known as P2Y6, belongs to the P2 receptor family and is activated by extracellular nucleotides. It undergoes variable splicing, resulting in multiple transcript variants that encode different protein isoforms [15]. Recent evidence suggests that P2RY6 signaling plays a role in regulating inflammation [16, 17]. Specifically, it has been found to have significant pro-inflammatory and pro-atherogenic roles in macrophages. Under inflammatory conditions, P2RY6 is involved in regulating neutrophil migration by controlling TLR2-induced IL-8 release from human monocytes [18, 19]. Furthermore, studies have demonstrated that P2RY6 receptors can contribute to host defense against bacterial infections, suggesting that they may act as novel mediators in enhancing the innate immune response to invading pathogens [20]. While previous research has investigated the role of P2RY6 in pancreatic cancer [21] and colorectal cancer [22], there is currently no corresponding article exploring its role in LUAD.

It is noteworthy to mention that this study is the first extensive analysis of P2RY6 expression and its correlation with various malignancies, including LUAD. By further investigating the association between P2RY6 expression and the prognosis of LUAD, the study has confirmed its findings through immunohistochemistry. The results of this study provide novel insights into the function of P2RY6 in LUAD and elucidate the intrinsic mechanisms by which P2RY6 influences the tumor microenvironment. Furthermore, these findings underscore the potential utility of P2RY6 in tumor immunotherapy applications.

## Methods

### Clinical specimens

In this study, twenty patients with LUAD were included, and samples of cancer tissue and adjacent normal paracancerous tissue were collected. It is crucial to note that these patients were diagnosed with LUAD based on pathological examination and had not undergone any prior radiotherapy or chemotherapy treatments. Prior informed consent was obtained from all patients, indicating their voluntary participation in the study. To ensure the ethical conduct of the research, the study protocol underwent review and approval by the Ethics and Anthropology Committee. All experiments and methods were performed in accordance with applicable standards and regulations, thereby guaranteeing the integrity and reliability of the study results.

### Data collection

We downloaded The Cancer Genome Atlas (TCGA) [23], genotypic tissue expression (GTEx) [24], RNA expression information, and clinical data from the UCSC Xena database [25] (https://xenabrowser.net/datapages/), which included more than 1000 tumor samples from 33 human cancers. We downloaded DNA copy number and methylation data from the cBioPortal database [26] (https://www.cbioportal.org/), and we ensured that the acquisition and application methods were consistent with relevant guidelines and regulations.

### Single-cell transcriptome sequencing data acquisition and analysis

The single-cell sequencing datasets were derived from GEO database (GSE131907). We obtained processed and cell annotated data of 11 LUAD primary tumor. All samples were sequenced using the Hiseq X10 (Illumina, San Diego, CA) with standard parameters. R (version 3.5.2) and Seurat R package (version 3.1.1) were used for quality control (QC) and analysis.

### Differential expression analysis of P2RY6

We used SangerBox (http://vip.sangerbox.com/home.html) software to compare the difference between P2RY6 in cancer tissues and normal tissues base on TCGA data, and the normal samples from the GTEx database were added to compare the differences of P2RY6 expression in cancer samples and normal paracancerous samples.

### Analysis of genetic alterations

Gene alteration characteristics of P2RY6 were queried by using the cBioPortal network (https://www.cbioportal.org/). After selecting the “Quick Select” as well as “TCGA Pan-Cancer Atlas Studies” options, information about the frequency of alterations, copy number alteration, and their mutation type were described in the “Cancer Types Summary” section of the TCGA database.

### Immunomodulators

To elucidate tumor-immune system interactions, we searched the online comprehensive database TISIDB (http://cis.hku.hk/TISIDB/) for immunosuppressants and immunostimulants significantly associated with P2RY6-related gene expression (Spearman correlation test, *p* < 0.05).

### Survival analysis

Kaplan–Meier analysis was performed to show the survival of patients in the high and low P2RY6 expression groups and to assess the potential of P2RY6 as a prognostic marker. Survivor, glmnet [27], survivor ROC, and survminer were the R packages used in the operations. To explore the relationship between P2RY6 expression and disease-specific survival (DSS), disease-free survival (DFS), overall survival (OS), and progression-free survival (PFS) in patients with each cancer type, we performed Cox regression analysis using TCGA in an R environment.

### Immune infiltration analysis

We explored the correlation between P2RY6 expression and immune infiltration by using the TIMER2 database and the CIBERSORT method. P2RY6 expression was also analyzed at the following cellular abundances, including B cells, CD4 + T cells, CD8 + T cells, neutrophils, dendritic cells (DCs), and macrophages. We compared their expression with P2RY6 after obtaining stromal score, ESTIMATE score, and immune score for each tumor type sample in TCGA by using ESTIMATE and summarized their relationship.

### Genome enrichment analysis

We identified the pathways associated with P2RY6 by gene set enrichment analysis (GSEA) by the cluster Profiler R package. Fold changes in mean gene expression between patients with high and low P2RY6 expression were sorted, and the sorted gene list was then represented in the input file, and assessed pathways by using HALLMARK pathways and Kyoto Encyclopedia of Genes and Genomes (KEGG) pathways.

### Construction of nomogram

After combining the clinical characteristics and risk scores of the patients, we made a nomogram of the cancer prognosis, which was created by the rms package of the R software. In order to predict the deviation of the probability from the actual situation, we display it by applying the bootstrap method (1000 repetitions) and using a calibration curve.

### Statistical analysis

P2RY6 high and low expression groups were defined according to TPM expression values. We used Spearman correlation test to mine the relationship between P2RY6 expression and targets such as tumor mutation burden and microsatellite instability. We calculated log-rank *p* values and HR values in survival analysis by COX regression analysis. *p* values were considered statistically significant when they were less than 0.05.

### Immunohistochemical staining

Tissue microarrays were prepared using tumor and paracancerous samples from surgical specimens. An experienced pathologist selected typical wax blocks of cancer and paraneoplastic tissues and marked representative lesions according to the sections. Holes were then punched into the wax blocks using a 1 mm diameter and 4 mm long tissue microarrayer. The tissue from the perforated wax blocks was accurately placed into small holes of empty white wax blocks. The resulting tissue microarray was subjected to baking and bleaching using a tissue section machine until all tissue specimens were planted in the empty white wax blocks. Finally, the tissue microarrays were baked at 70 °C, dewaxed in xylene, and hydrated in gradient ethanol. The routinely dewaxed tissue microarrays were subjected to high-temperature antigen repair at 100 °C for 2.5 min, with an additional 15 min of boiling while maintaining the fire. Immunohistochemical staining was performed according to the kit instructions (CK0062-20 T, Signalway Antibody, USA). Subsequently, the sections were incubated with 30% hydrogen peroxide for 15 min and immersed in 5% bovine serum albumin for 30 min at 37 °C to inhibit and block endogenous peroxidase activity. The tissue microarrays were then incubated overnight at 4 °C with anti-P2RY6 (dilution 1:300, Signalway Antibody, USA). After re-warming for 30 min, the sections were rinsed three times in phosphate-buffered solution (PBS), titrated with murine rabbit general secondary antibody, and stained with DAB. The sections were then restained with hematoxylin and sealed.

### Cell communication analysis

The identifying and illustrating alterations in the intercellular signaling network (iTALK) R package is a novel tool for intercellular communication analysis based on scRNA-seq [28], which captures high levels of down- or upregulated ligand-receptor gene pairs. We applied iTALK to analyze the differences in communication between cells with high versus low P2RY6 expression (cut-off TPM > 1). Differential analysis was performed using MAST [29]. Ligands were classified into four groups, namely cytokines/chemokines, immune checkpoint genes, and growth factors.

### Weighted gene co-expression network analysis

WGCNA is a method used to analyze the expression patterns of genes in multiple samples, which focuses on grouping genes with highly similar expression patterns. It focuses on grouping genes with highly similar expression patterns into modules and further analyzes the intrinsic associations between the module or key genes in the module and the features [30]. Genes with a mean expression value (TPM) greater than 1 are selected to construct the WGCNA network. The weighted gene co-expression network requires the selection of a soft thresholding power to make the relationships between genes in the co-expression network conform to the scale-free network distribution, making the correlation values more consistent with scale-free network characteristics and more biologically meaningful; the “pickSoftThreshold” function was applied for the soft threshold beta screening process. After obtaining the expression matrix and the best beta value, the “blockwiseModules” function of the WGCNA package was used for co-expression matrix construction. The association between gene modules and clinical features was judged by module significance (MS), which is the mean value of the significance of all genes in a module, and the higher the value, the stronger the association between the module and clinical features [31]. The higher the MS value, the stronger the association between the module and the clinical features, which can be used as a significant module for in-depth analysis.

## Results

### Increased expression of P2RY6 in pan-cancer

**Fig. 1:**
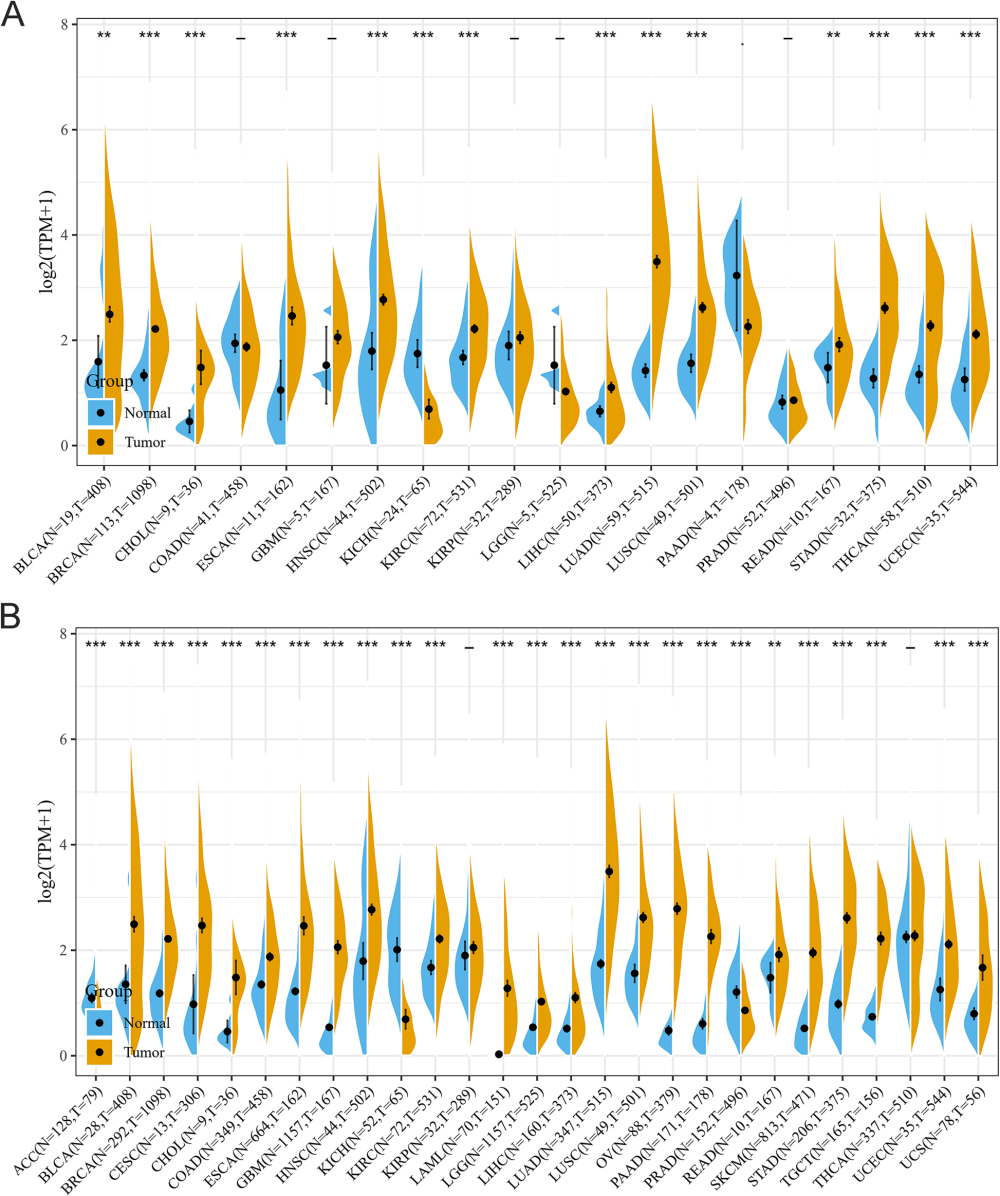
P2RY6 expression level in different human cancers. **A** The pan-cancer expression level of P2RY6 from TCGA database. Tumor tissues are represented by yellow spindles, and normal tissues are represented by blue spindles (*, *p* < 0.05; **, *p* < 0.01; and ***, *p* < 0.001). **B** The pan-cancer expression level of P2RY6 from TCGA and GTEx databases. Tumor tissues are represented by yellow spindles, and normal tissues are represented by blue spindles (*, *p* < 0.05; **, *p* < 0.01; and ***, *p* < 0.001)

Based on the data obtained from the pan-cancer database, we conducted an evaluation of the mRNA expression level of P2RY6. By comparing the expression level of P2RY6 in cancer and paracancerous tissues, we observed a significant difference between the two. The expression of P2RY6 mRNA was found to be significantly higher in most tumor tissues compared to normal tissues in various types of cancers including BLCA (bladder urothelial carcinoma), ESCA (esophageal carcinoma), CHOL (cholangiocarcinoma), BRCA (breast invasive carcinoma), LIHC (liver hepatocellular carcinoma), HNSC (head and neck squamous cell carcinoma), KIRC (kidney renal clear cell carcinoma), LUAD (lung adenocarcinoma), LUSC (lung squamous cell carcinoma), READ (rectum adenocarcinoma), THCA (thyroid carcinoma), STAD (stomach adenocarcinoma), and UCEC (uterine corpus endometrial carcinoma). The only exception was KICH, where P2RY6 expression showed a significant decrease. Considering the limited number of normal samples in the TCGA database, we decided to further analyze the expression level of P2RY6 in different types of cancers using TCGA’s tumor tissue data and the normal tissue data from the GTEx database. Our analysis included 27 types of tumors. The results revealed that apart from the aforementioned tumors, P2RY6 was also highly expressed in CESC (cervical squamous cell carcinoma and endocervical adenocarcinoma), GBM (glioblastoma multiforme), COAD (colon adenocarcinoma), LAML (acute myeloid leukemia), PAAD (pancreatic adenocarcinoma), OV (ovarian serous cystadenocarcinoma), LGG (brain lower grade glioma), PRAD (prostate adenocarcinoma), SKCM (skin cutaneous melanoma), and TGCT (testicular germ cell tumors) (Fig. 1B, all *p* < 0.01). These findings suggest that the differential expression of P2RY6 may be associated with the occurrence and development of various types of tumors, and it could potentially promote tumor growth, playing a significant role in tumorigenesis.

### The expression of P2RY6 is proportional to the stage of tumor

Using the “pathological staging” module of the World Health Organization, we conducted an analysis to examine the correlation between the expression of P2RY6 and the pathological stage of cancer. Our findings revealed a significant increase in the expression level of P2RY6 in stages III–IV of BLCA (bladder urothelial carcinoma) and THCA (thyroid carcinoma) (Supplementary Fig. 1). Interestingly, although there was an increasing trend in the expression of P2RY6 with higher tumor stages, such as KICH (kidney chromophobe), KIRC (kidney renal clear cell carcinoma), LUSC (lung squamous cell carcinoma), KIRP (kidney renal papillary cell carcinoma), MESO (mesothelioma), LUAD (lung adenocarcinoma), SKCM (skin cutaneous melanoma), TGCT (testicular germ cell tumors), and UVM (uveal melanoma), STAD (stomach adenocarcinoma), the differences were not statistically significant (Supplementary Fig. 1). Although there appears to be a correlation between P2RY6 expression and tumor stage, further evidence is required to clarify the relationship between P2RY6 and tumor progression.

### P2RY6 has clinical prognostic significance

**Fig. 2:**
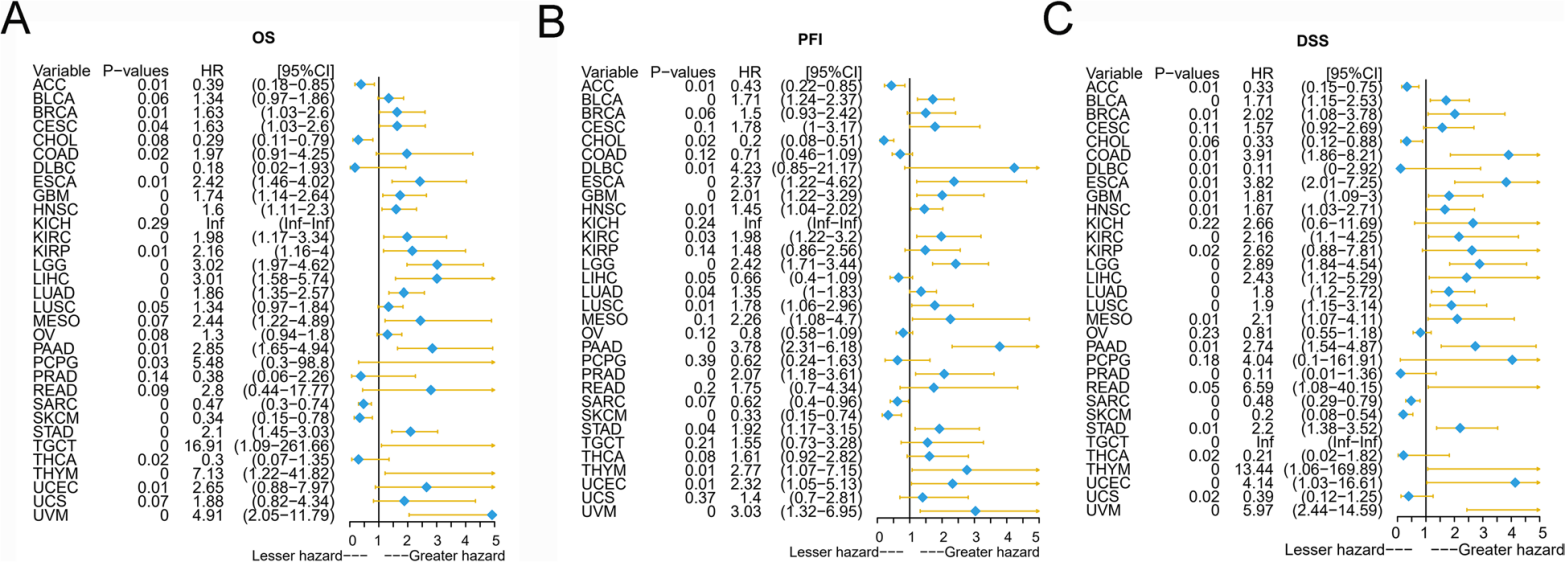
Forest plot for univariate Cox regression analysis of P2RY6. The univariate cox regression analysis results of P2RY6 on OS of TGCA pan-cancer patients are shown in **A**. The univariate cox regression analysis results of P2RY6 on the PFI of TGCA pan-cancer patients are shown in **B**. The results of univariate cox regression analysis of P2RY6 on DSS of TGCA pan-cancer patients are shown in **C**. Red represents a significant result

We utilized the Kaplan–Meier plotter database and conducted univariate Cox regression analysis to investigate the impact of P2RY6 expression on cancer patient survival. The results from Kaplan–Meier analysis revealed a significant correlation between high P2RY6 expression and a decline in overall survival (OS) for various cancer types, including CESC, BRCA, GBM, COAD, ESCA, KIRC, HNSC, KIRP, LIHC, LGG, LUAD, PAAD, PCPG (pheochromocytoma and paraganglioma), TGCT, THYM (thymoma), STAD, UCEC, and UVM (Supplementary Fig. 2, all *p* < 0.05). Additionally, we observed a significant association between decreased progression-free interval (PFI) and high P2RY6 expression in BLCA, DLBC, ESCA, GBM, HNSC, KIRC, LUSC, LGG, LUAD, PAAD, PRAD, TYM, STAD, UCEC, and UVM (Supplementary Fig. 3, all *p* < 0.05). Furthermore, high P2RY6 expression was found to be significantly associated with disease-specific survival (DSS) in BRCA, BLCA, COAD, HNSC, GBM, ESCA, KIRC, LUSC, KIRP, LGG, LIHC, LUAD, MESO, PAAD, TCGT, STAD, THYM, UCEC, and UVM (Supplementary Fig. 4, all *p* < 0.05). The results of Cox regression analysis indicated that elevated P2RY6 expression was closely related to poor OS, PFI, and DSS, suggesting that it may serve as a risk factor for most tumor patients (Fig. 2, all *p* < 0.05). Based on the aforementioned findings, we speculate that P2RY6 expression is an important factor influencing cancer patient survival, and the high expression of P2RY6 may be closely associated with unfavorable prognosis in various cancers.

### Gene set enrichment analysis of P2RY6

**Fig. 3:**
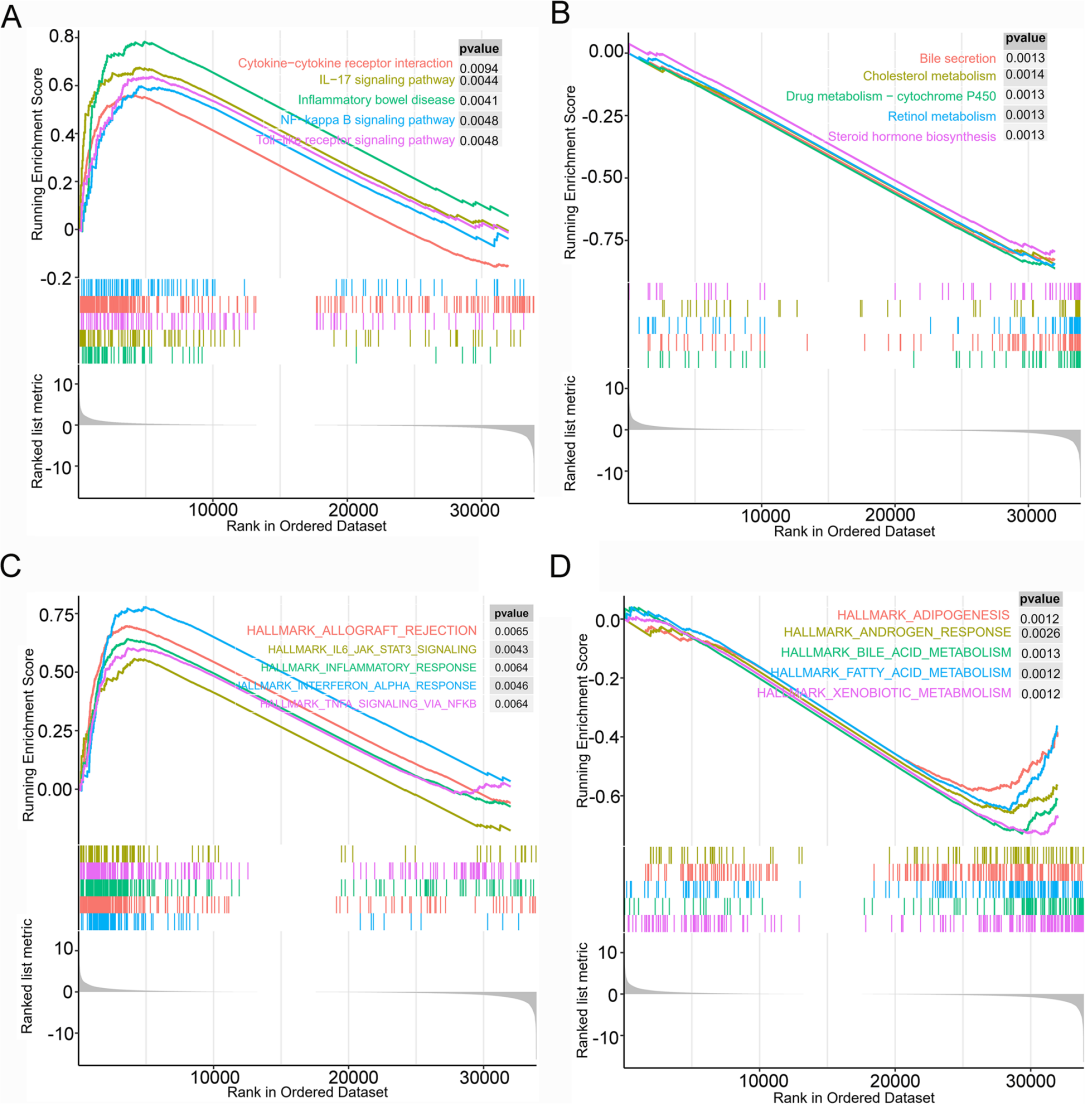
The results of gene set enrichment analysis of P2RY6 high expression group and low expression group through different ways. **A** The enrichment analysis results of the P2RY6 high expression group in the KEGG pathway. **B** The enrichment analysis results of the P2RY6 low expression group in the KEGG pathway. **C** The enrichment analysis results of the P2RY6 high expression group in the HALLMARK pathway. **D** The enrichment analysis results of the P2RY6 low expression group in the HALLMARK pathway. *p* < 0.05 was considered significant

To investigate the potential molecular functions of P2RY6 in various cancers, we performed gene cluster enrichment analysis. The results of the KEGG signaling pathway enrichment analysis indicated that the high expression group of P2RY6 was significantly enriched in pathways such as the IL-17 signaling pathway, cytokine-cytokine receptor interaction, inflammatory bowel disease, toll-like receptor signaling pathway, and NF-kappa B signaling pathway (Fig. 3A–B, all *p* < 0.01). Conversely, the low expression group was significantly enriched in pathways related to bile secretion, drug metabolism-cytochrome P450, cholesterol metabolism, steroid hormone biosynthesis, and retinol metabolism. These findings suggest that high P2RY6 expression is closely associated with various immune-related signaling pathways. Additionally, we verified these results using HALLMARK pathway analysis. It was observed that the high expression group of P2RY6 was significantly enriched in pathways such as allograft rejection, TNF-α signaling via NF-kB, interferon-alpha response, inflammatory response, and IL6/JAK/STAT3 signaling (Fig. 3C–D, all *p* < 0.01). On the other hand, the low expression group was significantly enriched in pathways related to adipogenesis, fatty acid metabolism, androgen response, xenobiotic metabolism, bile acid metabolism, and others. These findings further support the association between P2RY6 and immune-related signaling pathways, suggesting that P2RY6 may play a role in the immune response during the development and progression of different cancers.

### Analysis of immune cell infiltration and related immune regulatory genes and chemokines in P2RY6

Considering the growing recognition of the correlation between the tumor microenvironment, especially immunological characteristics, and the prognosis of various cancers, we conducted an analysis using the “CIBERSORT” method to assess the correlation of 22 immune cells. Our findings revealed a significant relationship between the expression of P2RY6 and the infiltration levels of CD4 memory resting T cells, Treg cells, and M2 macrophages in these immune cells across most tumors (Supplementary Fig. 5, all *p* < 0.01). These findings indicate that P2RY6 might be involved in the immune response of different cancers by promoting the infiltration of specific immune cells, which could potentially explain its cancer-promoting effect in most tumor types. Additionally, we explored the immune effects of P2RY6 in cancer from other perspectives. We analyzed the relationship between P2RY6 and immunosuppressive genes, immune-activating genes, chemokines, and chemokine receptors. Our analysis revealed a positive association between the expression of P2RY6 and immunosuppressive genes such as CD86, CD80, IL2RA, TGFB1, and LGALS9 in pan-cancer (Supplementary Fig. 6). Moreover, P2RY6 expression showed a positive correlation with chemokines and chemokine receptors such as CCL5, CCL8, and their corresponding receptors CCR5 and CCR8 (Supplementary Fig. 6 C–D). Furthermore, we observed a strong correlation between the expression of P2RY6 and StromalScore, ImmuneScore, and ESTIMATEScore in most tumors, demonstrating a significant positive association (Supplementary Fig. 7, 8 and 9, all *p* < 0.05). Based on these results, we speculate that P2RY6 might play a role in the chemotactic recruitment of immune cells and the regulation of immune signaling pathways in the tumor immune response.

### Correlation between P2RY6 and immune checkpoint genes, TMB, and MSI in human cancer

Immune checkpoint genes play a crucial role in tumor immunotherapy. To further investigate the potential of P2RY6 in clinical immunotherapy, we analyzed its correlation with immune checkpoint genes. Our analysis encompassed 47 common immune checkpoint genes, and we observed a strong correlation between the expression of P2RY6 and these genes in various types of cancers (Supplementary Fig. 10). This indicates that P2RY6 might coordinate the activity of immune checkpoint genes in different signal transduction pathways, making it a promising target for immunotherapy. Furthermore, we conducted a Spearman correlation test to explore the relationship between P2RY6, tumor mutational burden (TMB), and microsatellite instability (MSI). The results demonstrated a positive correlation between P2RY6 and TMB in BLCA, BRCA, GBM, ESCA, KIRP, LGG, LUAD, SARC, and THYM. Additionally, P2RY6 exhibited a positive correlation with MSI in BLCA, PAAD, COAD, BRCA, DLBC, KIRC, READ, HNSC, SKCM, STAD, and TGCT (Supplementary Fig. 11). These findings shed light on the role of P2RY6 in the immune response and immune mechanisms within the tumor microenvironment. The positive associations with TMB and MSI suggest that P2RY6 may influence the immune response in these specific cancer types. These results deepen our understanding of the potential implications of P2RY6 in the context of tumor immunotherapy.

### Expression level of P2RY6 in LUAD and its relationship with immune cells

**Fig. 4:**
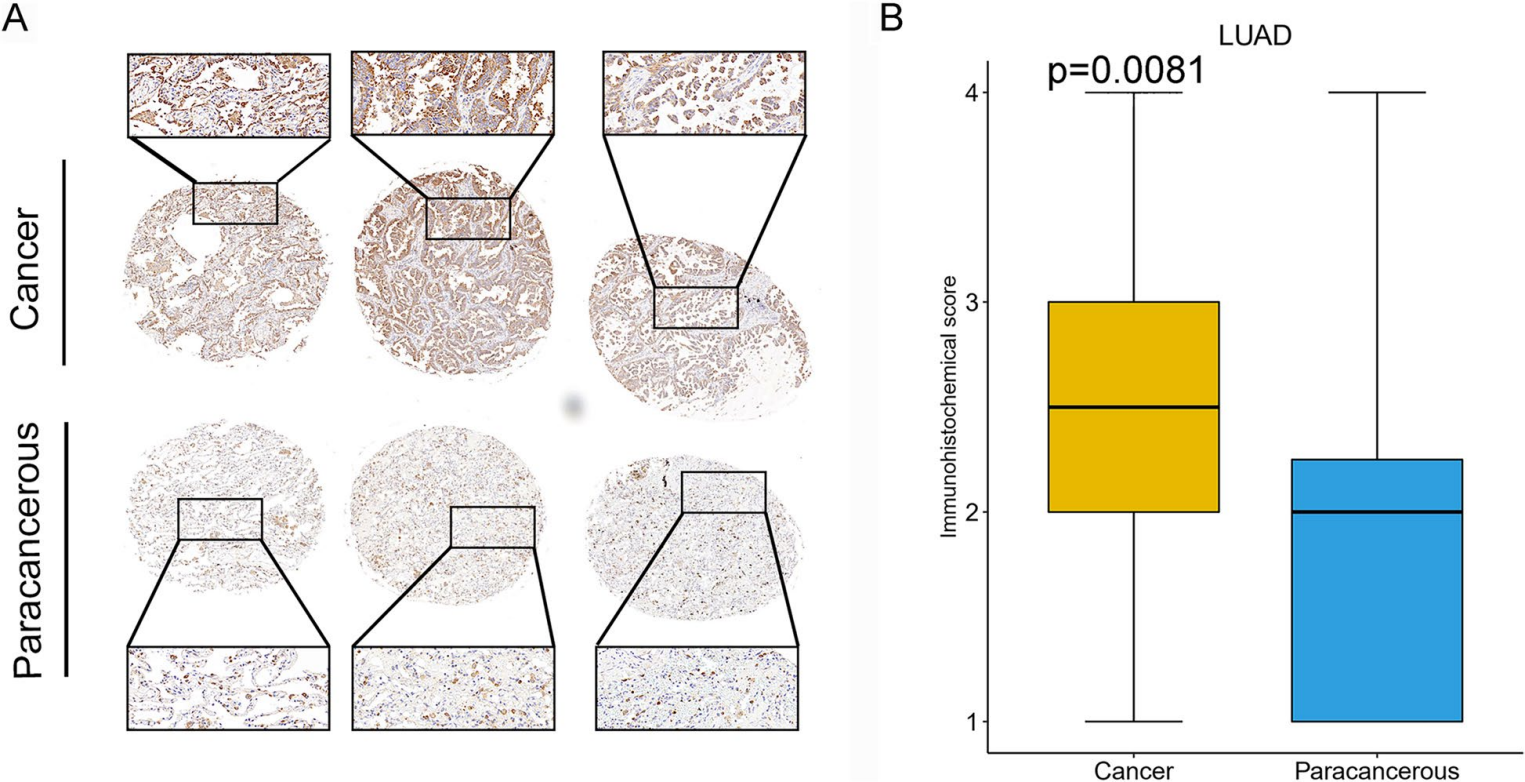
Analysis of the protein expression level of P2RY6. **A** Representative images of immunohistochemical detection of P2RY6 expression in LUAD. **B** Box plot of immunohistochemical scores related to LUAD and adjacent tissues

The data analysis revealed a significant difference in the expression level of P2RY6 in LUAD compared to other cancer tissues, with LUAD showing notably higher expression. This higher expression of P2RY6 in LUAD suggests its potential involvement in promoting the development of LUAD and its association with prognosis. Kaplan–Meier analysis and univariate Cox regression analysis further supported this observation, demonstrating a significant correlation between high P2RY6 expression and worsened overall survival (OS), progression-free interval (PFI), and disease-specific survival (DSS) in LUAD patients. To delve deeper into the role of P2RY6 in LUAD, we examined its protein expression levels using immunohistochemical staining. The staining of LUAD tissues showed that P2RY6 was predominantly located in the cell membrane and cytoplasm, with some cells displaying nuclear staining. Additionally, P2RY6 staining appeared darker in tumors compared to adjacent para-cancerous tissues (Fig. 4A). Moreover, based on corresponding immunohistochemical scores, the expression level of P2RY6 in LUAD was significantly higher than that in para-cancerous tissues (Fig. 4B, *p* = 0.0081). These experimental results align with the findings from the database analysis, reinforcing the notion that P2RY6 plays a pivotal role in LUAD. Further investigation into the signal pathways regulated by P2RY6 in the LUAD immune response revealed a range of immunomodulators, including IDO1, LGALS9, IL10, TIGIT, IL10RB, TNFRSF18, CD40, and IL2RA, which exhibited significant positive correlations with P2RY6 in LUAD (Supplementary Fig. 12, *p* < 0.01). Additionally, by utilizing the TCGA database, we assessed the level of immune cell infiltration in LUAD according to P2RY6 expression. Notably, the high P2RY6 expression group displayed lower levels of resting CD4 T cell infiltration (Supplementary Fig. 13A, *p* < 0.01), which is associated with poor OS in LUAD. Furthermore, the abundance of Treg cells was significantly increased in the high P2RY6 expression group (Supplementary Fig. 13B, *p* < 0.05). These findings collectively indicate that P2RY6 plays a crucial role in immune cell infiltration in LUAD and exerts varying degrees of influence on tumor occurrence and progression.

### Prognostic value and risk model of P2RY6-related immunomodulators in LUAD

The study employed univariate Cox regression analysis to investigate the prognostic significance of P2RY6-related immunomodulators in LUAD. Several genes, including PVRL2, TGFBR1, CD276, CD70, MICB, NT5E, and RAET1E, exhibited significant associations with P2RY6 (Supplementary Fig. 14A, all *p* < 0.05). Subsequently, multivariate Cox regression analysis was conducted on the genes that demonstrated statistical significance in the univariate analysis. This analysis identified several genes, namely TGFBR1, CD70, CD276, NT5E, MICB, and RAET1E, which were closely correlated with poor prognosis in LUAD (Supplementary Fig. 14B, all *p* < 0.05). Based on these findings, a related prognostic risk model was constructed by calculating the gene expression values and coefficients, which were then used to calculate the risk scores for patients with LUAD. The correlation between these genes and overall survival (OS) was further evaluated using the Kaplan–Meier plotter database. The results revealed that patients with a high-risk score exhibited significantly lower survival time and worse OS (Supplementary Fig. 14C, *p* < 0.01). Moreover, the corresponding receiver operating characteristic (ROC) curve analysis demonstrated that the area under the curve (AUC) for the risk score was 0.807, while the AUC for the TNM staging was 0.683. Importantly, when combining the two, the AUC value increased to 0.83, indicating that the P2RY6-based prognostic model possessed superior discrimination capabilities compared to TNM staging (Supplementary Fig. 14D).

### Prognostic value of P2RY6-related risk models in LUAD cohort

**Fig. 5:**
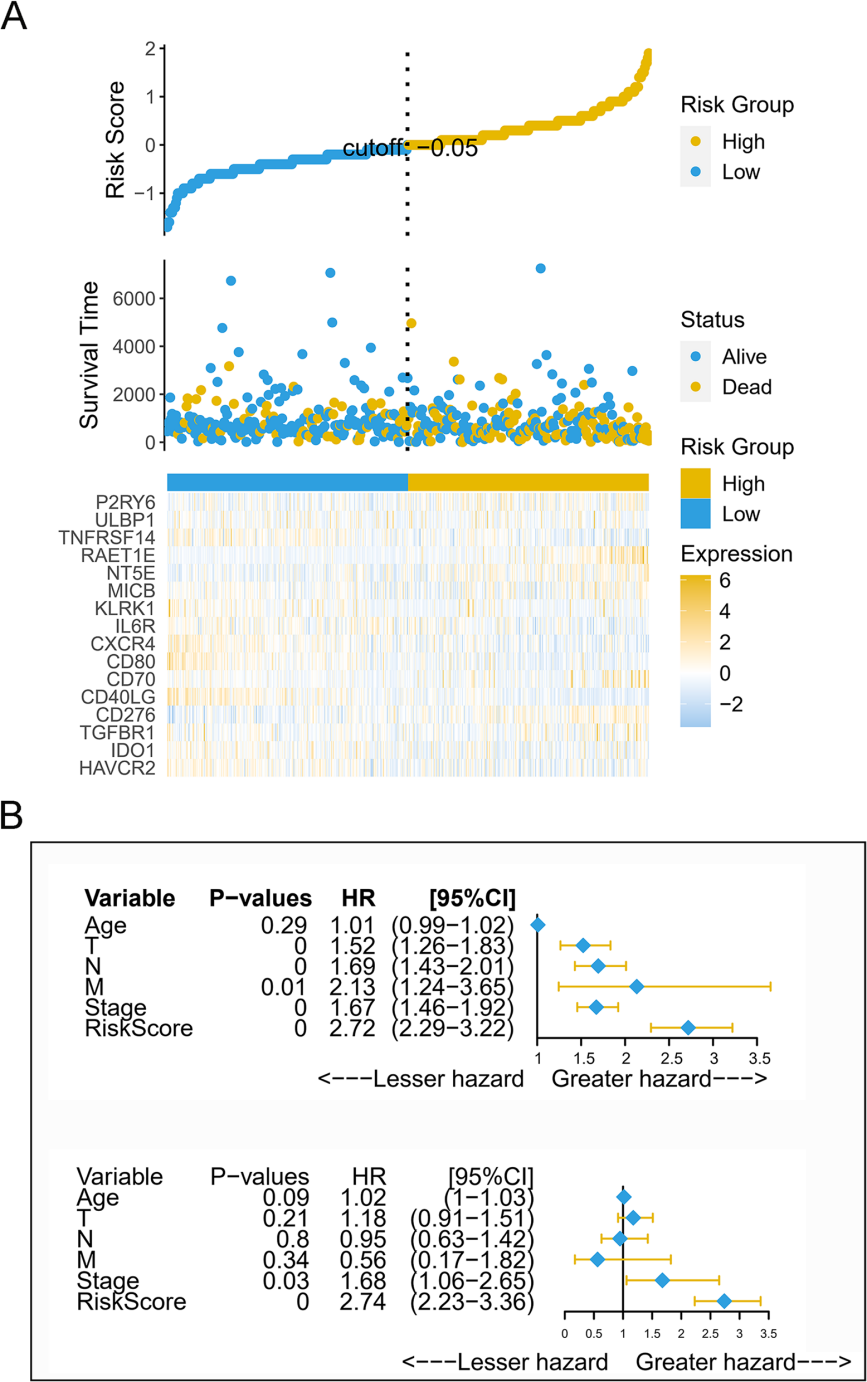
Analysis of the prognostic value of the risk scoring model in LUAD patients. **A** The risk score distribution, patient’s survival status, and related heat map of gene expression profile for LUAD. **B** The results of univariate and multivariate Cox regression analysis on the overall survival risk score of LUAD

To explore the accuracy and prognostic value of the risk model, the study utilized correlation heat maps and conducted Cox regression analysis. Figure 5A depicted the distribution of risk scores, survival time, and gene expression profiles in LUAD. The chart clearly demonstrated that higher risk scores were associated with shorter survival time and higher mortality rates. Notably, it was evident from the figure that the expression of the P2RY6 gene increased as the risk score increased. In both univariate and multivariate Cox regression analyses shown in Fig. 5B (all *p* < 0.05), the risk score was found to be significantly correlated with the survival rate of LUAD patients. It emerged as an independent prognostic factor, exerting a stronger impact on the survival rate compared to age and stage. Therefore, the risk score based on the P2RY6 gene exhibited favorable prognostic performance and could serve as an independent prognostic index for LUAD.

### Construction of P2RY6-related nomogram

**Fig. 6:**
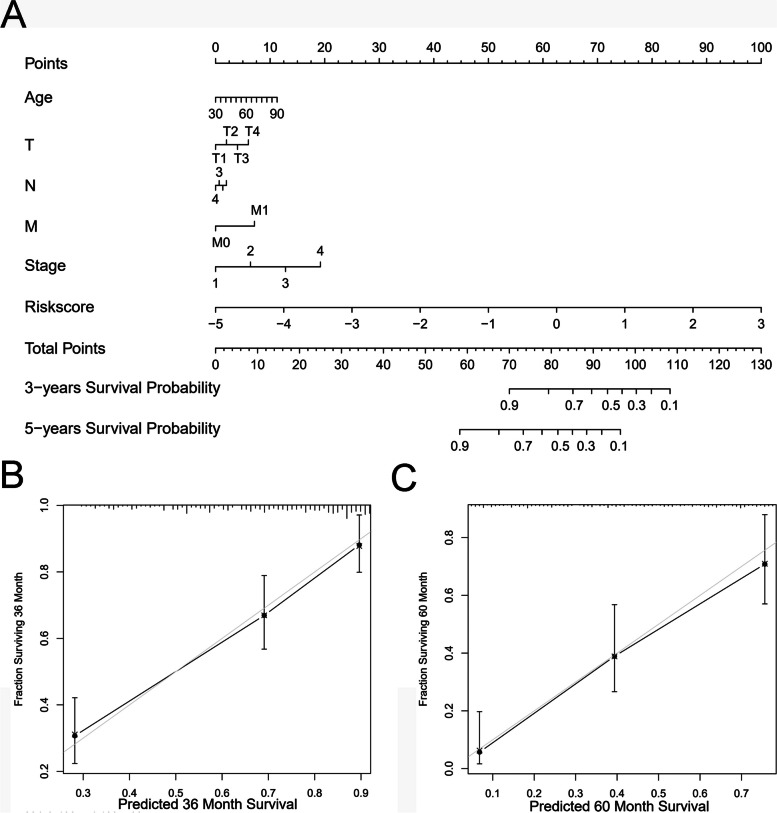
The establishment of a nomogram related to the risk score is used for the prognosis of LUAD patients. **A** A nomogram that can be used to predict the 3-year and 5-year survival rates of LUAD patients. **B** A calibration curve used to calibrate the 3-year survival rate of the nomogram related to LUAD patients. **C** A calibration curve used to calibrate the 5-year survival rate of the nomogram related to LUAD patients

Finally, we constructed a prognostic nomogram for LUAD (Fig. 6A), which could be used to predict the 3-year and 5-year survival probability of related individuals. From the calibration curve, it could be seen that the predicted survival probability of the nomogram for 3 and 5 years was in good agreement with the ideal reference line, which was very close to the ideal 45 degree (Fig. 6B–C).

### Single-cell transcriptome analysis of the P2RY6 in the LUAD tumor microenvironment

**Fig. 7:**
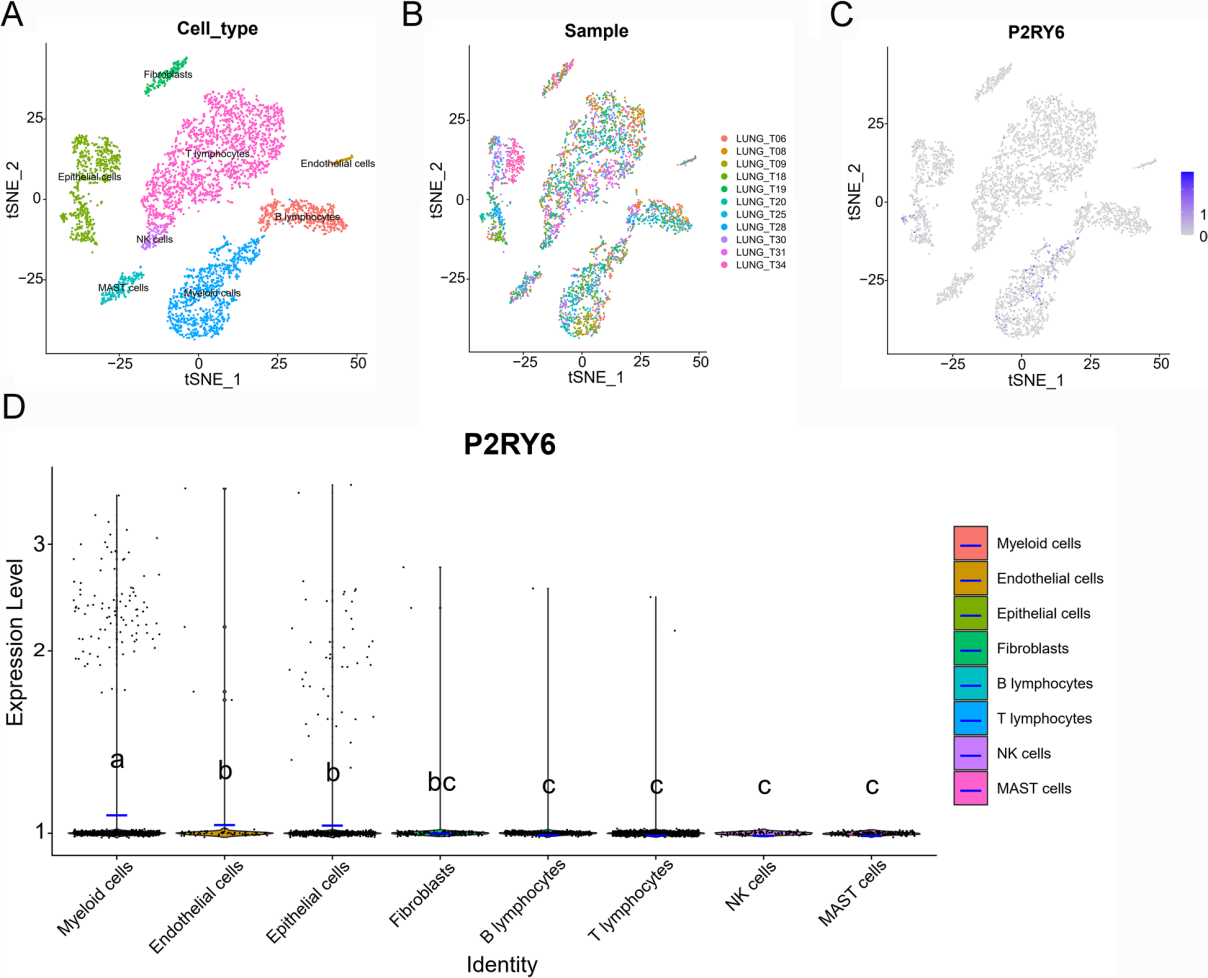
Single-cell transcriptomic atlas of LUAD. **A** A tSEN plot of LUAD samples with eight distinct cell types. **B** A tSEN plot of LUAD from 11 different samples. **C** A tSEN plot of P2RY6 in different cell types. **D** Comparison of P2RY6 in different tumor microenvironment cells. The blue horizontal line on the violin plot indicates the median of P2RY6 expression. The letters at the top indicate whether there is a statistical difference between the two cells. Different letters indicate that the difference is statistically significant

LUAD scRNA-seq data from 11 primary foci was obtained from the GEO database. Cell clustering analysis based on the t-distributed stochastic neighbor embedding (tSNE) algorithm showed that the above cells could be classified into eight clusters, namely T lymphocytes, endothelial cells, B lymphocytes, epithelial cells, NK cells, MAST cells, myeloid cells, and fibroblasts (Fig. 7A). Additionally, we found that all eight of these cells were present in samples from different sources (Fig. 7B). The expression of P2RY6 in the LUAD tumor microenvironment is cell-specific, being highest in myeloid cells and endothelial cells and almost absent in NK cells and MAST cells (Fig. 7C and D). It should be noted that this phenomenon can also be caused by bias in the inclusion of cells in scRNA-seq or by the lack of sensitivity of the sequencing method. Overall, the P2RY6 differed significantly among the different LUAD cell clusters, suggesting that P2RY6 may be a characteristic of LUAD cells.

**Fig. 8:**
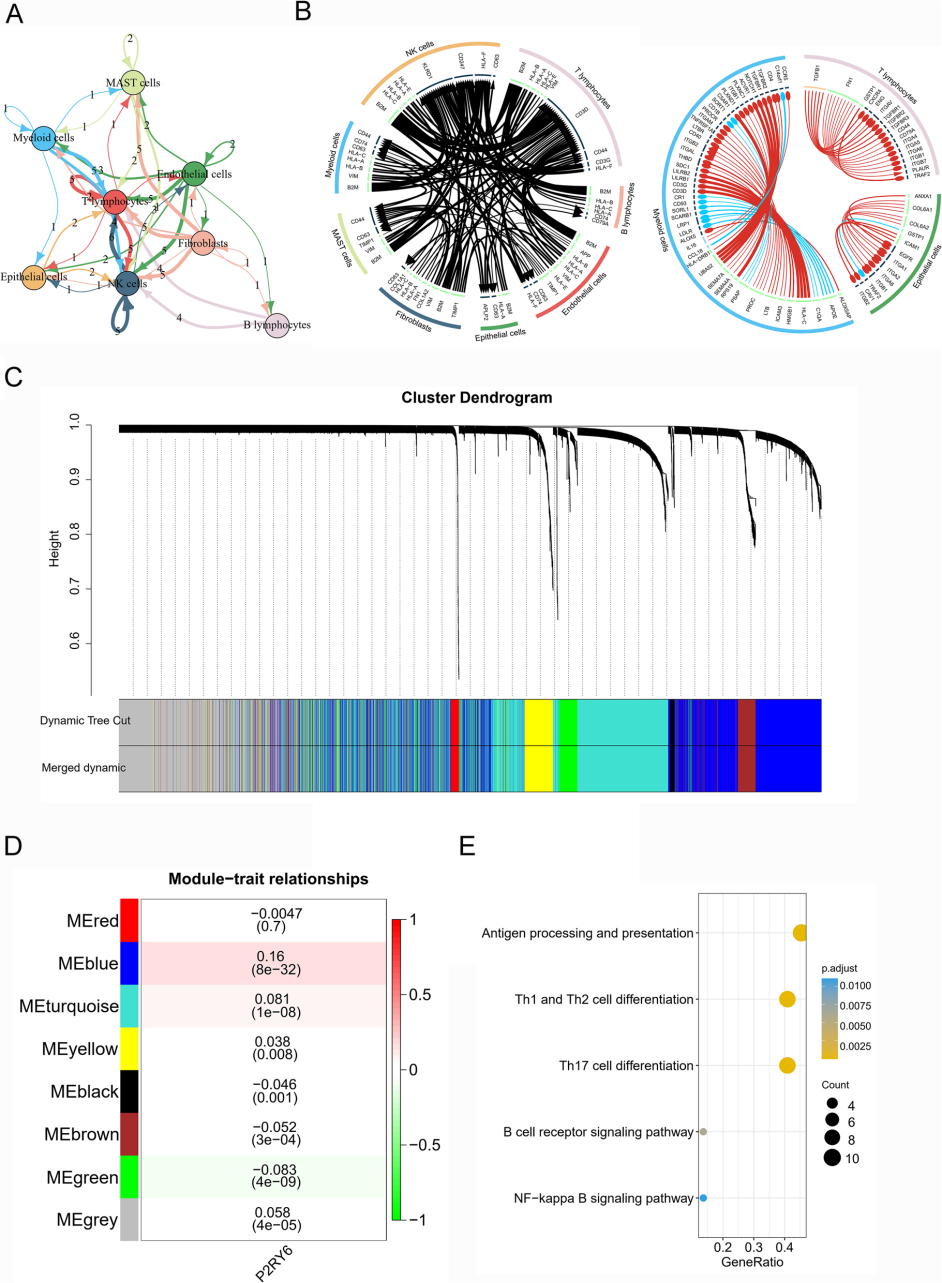
Cell communication analysis and WGCNA analysis of LUAD. **A** Network plot showing the number of ligand-receptor interactions detected in LUAD. **B** Circos plots showing top 100 highly expressed ligand-receptor interactions (upper panel). Circos plot showing significantly different ligand-receptor interactions between cells high and low P2RY6 expression (lower panel). **C** Clustering dendrograms of genes. **D** Gene module–trait associations. Each row corresponds to an ME. The corresponding correlation and *p*-value are shown in the cells. The cells are color coded by the correlation according to the color legend. **E** Significantly enriched KEGG pathway in model blue

To further explore the mechanisms of P2RY6 in the LUAD tumor microenvironment, we performed a cellular communication analysis. We first depict the top 100 receptor-ligand pairs with the highest abundance. We found close cellular communication between T lymphocytes, NK cells, and fibroblasts (Fig. 8A). CD3D was the main receptor responsible for cellular communication in T lymphocytes. KLRD1 was the main receptor responsible for cellular communication in NK cells. The B2M and HLA gene families were important ligands responsible for cellular communication in the cells mentioned above (Fig. 8B). Our analysis of cellular communication between high and low P2RY6 expressing cells identified a total of 102 statistically significant receptor-ligand pairs (Fig. 8B). Interestingly, we found that most of the ligands were significantly upregulated in the P2RY6 high expression group. For example, the ligand COL6A1, which is predominantly upregulated in epithelial cells (malignant cells), has been reported to promote bone metastasis in lung cancer [32]. The above results partly explain the potential mechanism for the poor prognosis of LUAD patients with high P2RY6 expression.

To clarify the gene modules regulated by P2RY6, we used WGCNA to identify P2RY6-related gene modules. The gene network was constructed using the “pickSoftThreshold” function to select *β* = 4 at “SFT.R.sq” of 0.90. The gene clustering results were further sheared according to the criteria of hybrid dynamic shearing tree by setting the lower limit of the number of gene modules to 30 to obtain different gene modules. After determining the gene modules, the eigenvector value ME was then calculated for each module. The ME represents the average level of gene expression values within each module. All modules were then re-clustered using the “cutreeDynamic” function to merge similar modules into new ones. The modules with high similarity were merged to obtain a total of 8 gene modules (Fig. 8C). The black module contained 42 genes, the blue module contained 1635 genes, the brown module contained 462 genes, the green module contained 294 genes, the grey module contained 1212 genes, the red module contained 72 genes, the turquoise module contained 1736 genes, and the yellow module contained 392 genes. It is important to note that the grey module is a mixed module; i.e., the genes contained in it cannot be combined into one module. We next calculated the gene modules associated with P2RY6 expression using Pearson correlation analysis of the correlation coefficients and corresponding *p*-values between the eigenvector gene ME and clinical features in the modules to identify significant modules. The results showed that the blue module was significantly positively correlated with P2RY6 expression (*p* = 8 × 10^−32^, Fig. 8D). We performed a pathway enrichment analysis of the genes in the blue module and the results showed that the blue module was significantly enriched in the pathways “Antigen processing and presentation” and Th1 and Th2 cell differentiation. The blue module was significantly enriched in “Antigen processing and presentation,” “Th1 and Th2 cell differentiation,” and other pathways (Fig. 8E). The above pathways are closely related to tumor immunity, suggesting that P2RY6 may serve as a potential target.

## Discussion

In our study, we first analyzed the expression level of P2RY6 from the perspective of pan-cancer, and found that compared with normal tissues, the expression level of P2RY6 in a variety of cancer tissues was significantly higher, and the results of Kaplan–Meier analysis and univariate Cox regression analysis showed that the poor prognosis of patients was closely correlated with the high expression of P2RY6, so we concluded that the high expression of P2RY6 in most tumors may not be conducive to the survival of patients with related tumors. Previous studies have pointed out that the progression of the disease is closely correlated with the expression of P2RY6, and there is a strong correlation between different levels of P2RY6 expression and different prognosis. For example, Wan et al. pointed out that P2RY6 is closely correlated with the prognosis and treatment of gastric cancer [33]. Qing et al. pointed out that the high expression of P2RY6 can be used as a prognostic indicator of pancreatic cancer [34]. Therefore, after analyzing several databases including GEPIA, Timer, Kaplan–Meier plotter, and TCGA, we speculate that P2RY6 may be a cancer-promoting gene, and its expression may be a valuable prognostic biomarker in cancer.

To further investigate the potential mechanisms of P2RY6 in various cancers, we analyzed the enrichment of P2RY6-related signaling pathways using the TCGA database and found significant correlations with multiple immune-related pathways. Our work also demonstrated a close relationship between P2RY6 gene expression and the degree of immune cell infiltration. Previous studies have shown that tumor-infiltrating lymphocytes can serve as a novel prognostic index [35] and be utilized for the treatment of solid tumors [36]. Interestingly, we found that P2RY6 is closely related to the immunity of many cancers, especially LUAD. Combined with the previously observed low infiltration of CD4 memory resting T cells and high infiltration of Treg cells in LUAD, we speculate that immune cell infiltration may play an extraordinary role in the formation and treatment of LUAD. It has been proved that these cells play an important role in innate immunity and participate in the function of immune cells [37–39]. Richard Kennedy et al. pointed out that CD4 + T memory cells can enhance anti-tumor response by enhancing clonal expansion of tumor site, preventing activation-induced cell death, and giving priority to the generation of immune memory cells by CTL as antigen presenting cells [40], which is consistent with the low infiltration level of CD4 memory resting T cells caused by high expression of P2RY6 in our study. In the process of tumorigenesis and development, Treg cells can inhibit the anti-tumor response, so a large number of Treg cells infiltrate into tumor tissues, which is often closely related to poor prognosis [38]. From these, we can also explain why the increased level of Treg cell infiltration in tumor microenvironment often leads to worse prognosis of patients with high expression of P2RY6. In addition, it is worth noting that when we explored the potential relationship between P2RY6 and immunomodulators, we found that the expression of this gene was positively related to a variety of immune activators and immunosuppressants, which means that P2RY6 may have a synergistic effect with other checkpoint members, thus playing a specific role in the regulation of tumor immune microenvironment.

Tumor microenvironment is an important part of tumor tissue, and its composition varies with tumor types, but its specific characteristics include blood vessels, stromal cells, immune cells, and extracellular matrix [41–44]. With the rapid development of tumor immunotherapy, there is a lot of evidence that tumor microenvironment has high application value in clinical treatment [44–47]. and now immunotherapy has been clinically selected for the treatment of a variety of tumors (such as renal cell carcinoma [48] and melanoma [49]). After analyzing the 47 immune checkpoint genes collected, we thought that P2RY6 may be a new target for immunotherapy, and after analyzing the relationship between P2RY6, TMB, and MSI, we understand that P2RY6 was positively related to TMB and MSI in most tumors. These results may provide a theoretical basis for combined molecular targeted immunotherapy in the future. Our understanding of the immunological characteristics of P2RY6 microenvironment makes us confident to infer that LUAD may affect tumor immunity and thus affect the prognosis of LUAD patients. Some genes of the same family with P2RY6 have developed corresponding immunotherapy [50]. Inspired by this, we think that P2RY6 may have a good prospect in the immune regulation of tumor microenvironment.

Furthermore, based on the immunomodulators genes related to P2RY6, we successfully constructed the relevant risk model by using Cox regression analysis. Finally, the predicted value of the risk model is verified by the nomogram. Our results show that the risk score is closely correlated with the survival rate of patients with LUAD, and may become an independent prognostic factor with high prognostic value. As a result, patients with early LUAD can be divided into high-risk and low-risk groups with significantly different overall survival rates. The risk scores obtained from P2RY6-related immunomodulators can distinguish the risk groups defined by different gene differential expression, which may be helpful for the further study of LUAD-related prognostic signals [51, 52]. Combining these results, we can find that the risk score is closely related to the immune microenvironment of LUAD, so we are more confident that P2RY6 will be a new target of LUAD immunotherapy and bring results different from traditional treatments in clinical treatment [53, 54].

Our research still has some limitations. Firstly, despite adhering to strict standards, individual variations among different cancer patients may introduce bias to our findings. Secondly, our analysis of P2RY6 using Cox regression may be influenced by other potential prognostic factors such as patients’ socioeconomic status and treatment plans, which are inherent disadvantages of database research. Importantly, this study is based on bioinformatics analysis, and the limitations of public databases may result in a lack of clinical data analysis for LUAD patients. Therefore, before applying our model to clinical practice, further in vivo and in vitro studies are necessary to confirm the interaction between P2RY6 and the LUAD tumor microenvironment.

## Conclusions

In summary, our findings suggest that P2RY6 holds promise as a novel prognostic marker for LUAD. We have also explored the potential role of P2RY6 in tumor immunology and its prognostic value in LUAD. Additionally, we developed a nomogram integrating independent clinical factors, which can accurately predict prognosis. Based on our results, we propose that P2RY6 may serve as a target for personalized immunotherapy in LUAD patients. We hope that these findings will provide valuable references for future laboratory research and clinical applications of P2RY6 in cancer prognosis and immunotherapy.

### Supplementary Information

**Additional file 1: Supplementary Figure 1. **The expression level of P2RY6 in different stages of tumor. (A–B). The logging scale uses Log2 (TPM+1), and analyzes the expression of P2RY6 gene through the TCGA database according to the main pathological stages (I-II, III-IV).

**Additional file 2: Supplementary Figure 2. **Kaplan-Meier survival curve of OS in difference of various tumor.

**Additional file 3: Supplementary Figure 3.** Kaplan-Meier survival curve of PFI in difference of various tumor types.

**Additional file 4: Supplementary Figure 4. **Kaplan-Meier survival curve of DSS in difference of various tumor types.

**Additional file 5: Supplementary Figure 5. **The results of correlation analysis between the expression of P2RY6 in pan-cancer and the impact of immune microenvironment are related to the level of immune cell infiltration. Positive correlation is represented by yellow, and negative correlation is represented by blue, and the darker the color, the stronger the correlation. *, *p* <0.05, **, *p* <0.01, and ***, *p* <0.001.

**Additional file 6: Supplementary Figure 6. **Correlation analysis between P2RY6 expression and immune regulation-related genes. (A) Heat map of the correlation between P2RY6 expression in pan-cancer and immune activation genes. (B) The heat map of the correlation between the expression of P2RY6 in pan-cancer and the immunosuppressive state-related genes. (C) Heat map of the correlation between the expression of P2RY6 in pan-cancer and chemokine genes. (D) Heat map of the correlation between the expression of P2RY6 in pan-carcinoma and chemokine receptor genes. Positive correlation is represented by yellow, and negative correlation is represented by blue, and the darker the color, the stronger the correlation. *, *p* <0.05, **, *p* <0.01, and ***, *p*<0.001.

**Additional file 7: Supplementary Figure 7. **Results of correlation analysis between P2RY6 expression and Stromal Score in pan-cancer.

**Additional file 8: Supplementary Figure 8. **Results of correlation analysis between P2RY6 expression and Immune Score in pan-cancer.

**Additional file 9: Supplementary Figure 9. **Results of correlation analysis between P2RY6 expression and ESTIMATE Score in pan-cancer.

**Additional file 10: Supplementary Figure 10. **Correlation analysis between P2RY6 expression and pan-cancer immune marker set. *p<0.05; **p <0.01; *** *p* <0.001.

**Additional file 11: Supplementary Figure 11.** The relationship between P2RY6 expression and TMB and MSI in pan-cancer. (A) TMB. (B) MSI. TMB, tumor mutation burden; MSI, microsatellite instability. The radar chart shows Spearman's correlation coefficient and *p*-value.

**Additional file 12: Supplementary Figure 12. **Correlation analysis between P2RY6 gene and immunomodulator.

**Additional file 13: Supplementary Figure 13.** Relationship between P2RY6 expression level and tumor infiltrating immune cells in LUAD. (A) The level of resting T cell infiltration in CD4. (B) The infiltration level of Treg cells. *p <0.05; **p <0.01; *** *p* <0.001.

**Additional file 14: Supplementary Figure 14. **The construction of P2RY6 related risk model. (A) Forest plot results of univariate Cox regression analysis for patients with LUAD. (B) Forest plot results of multivariate Cox regression analysis for patients with LUAD. (C) Kaplan-Meier curve for predicting OS in patients with LUAD based on the TCGA data set. (D) Receiver operating characteristic curve (ROC) analysis results of patients with LUAD.

## Data Availability

The dataset supporting the conclusions of this article is included within the article. Xena database [23] (https://xenabrowser.net/datapages/), cBioPortal database [24] (https://www.cbioportal.org/), cBioPortal network (https://www.cbioportal.org/), and TISIDB (http://cis.hku.hk/TISIDB/).

## Abbreviations

LUAD: Lung adenocarcinoma
NSCLC: Non-small cell lung cancer
TCGA: The Cancer Genome Atlas
OS: Overall survival
DFS: Disease-free survival
DSS: Disease-specific survival
