# Supplementary figures and images for: Immunological role and clinical prognostic significance of P2RY6 in lung adenocarcinoma: a multi-omics studies and single-cell sequencing analysis
Source: World J Surg Oncol. 2023 Oct 26;21:341. doi: 10.1186/s12957-023-03216-1 (PMC10601148; doi:10.1186/s12957-023-03216-1)

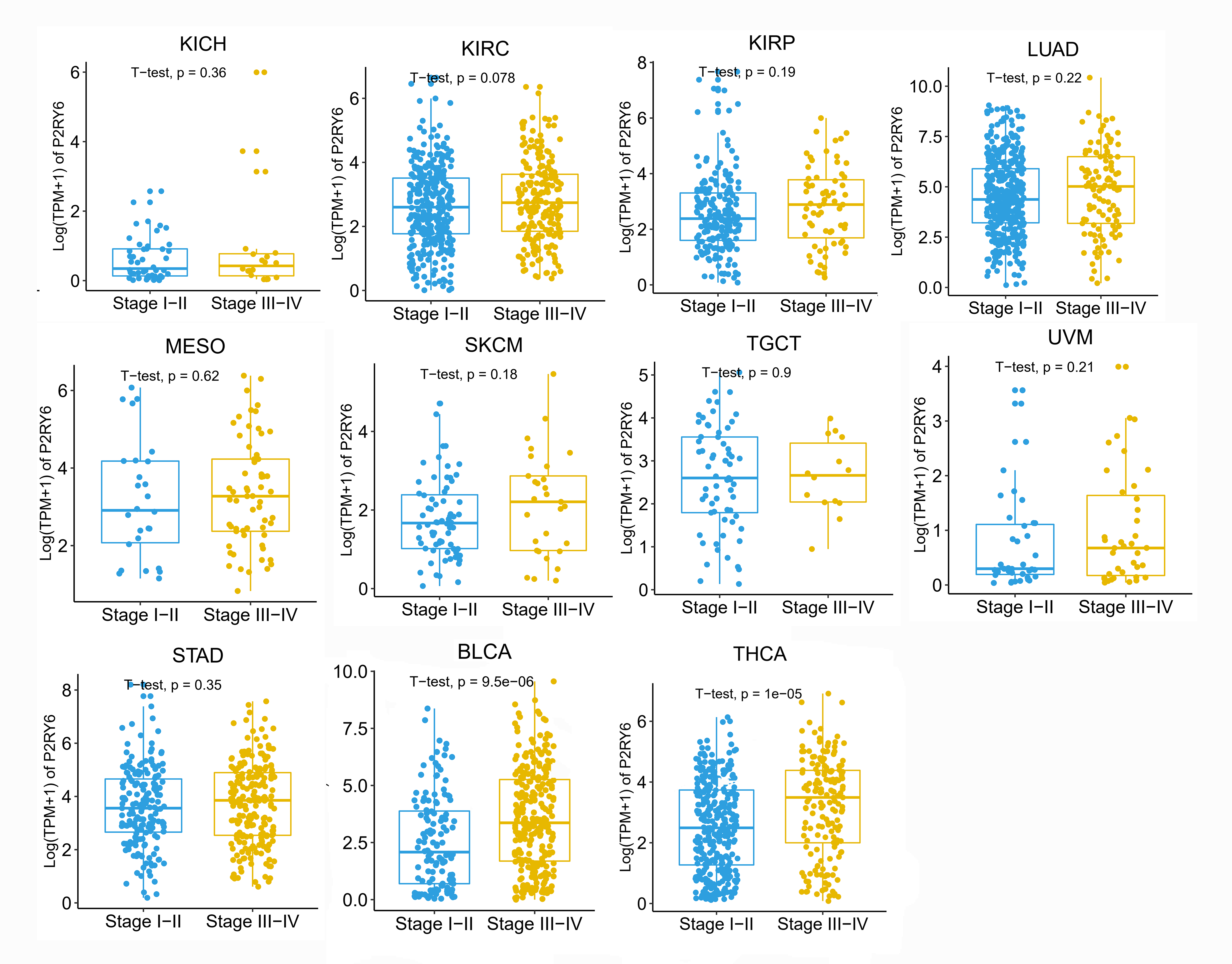

Supplement: Supplementary file 1 — Additional file 1: Supplementary Figure 1. The expression level of P2RY6 in different stages of tumor. (A–B). The logging scale uses Log2 (TPM+1), and analyzes the expression of P2RY6 gene through the TCGA database according to the main pathological stages (I-II, III-IV). [file 12957_2023_3216_MOESM1_ESM.tif]

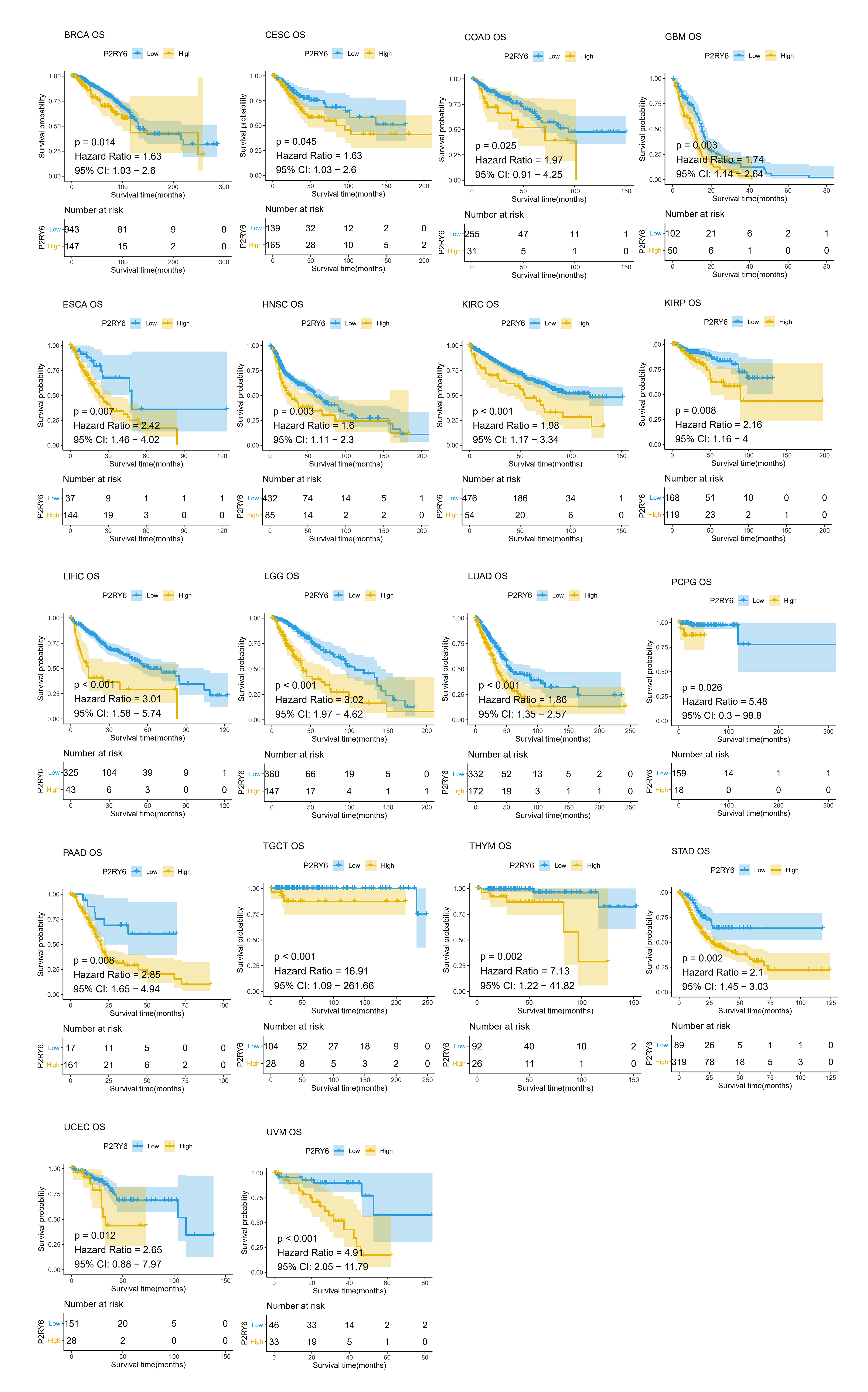

Supplement: Supplementary file 2 — Additional file 2: Supplementary Figure 2. Kaplan-Meier survival curve of OS in difference of various tumor. [file 12957_2023_3216_MOESM2_ESM.tif]

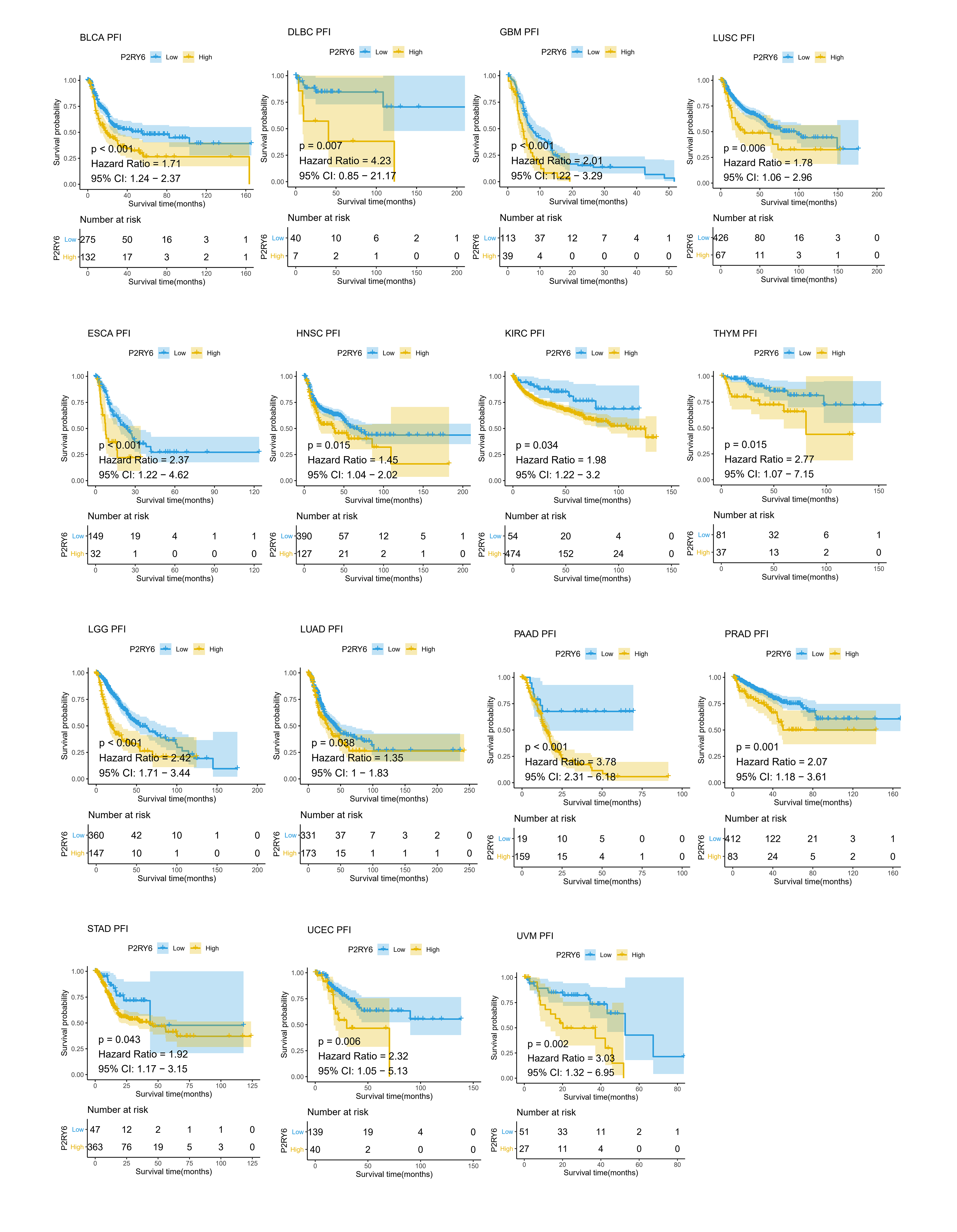

Supplement: Supplementary file 3 — Additional file 3: Supplementary Figure 3. Kaplan-Meier survival curve of PFI in difference of various tumor types. [file 12957_2023_3216_MOESM3_ESM.tif]

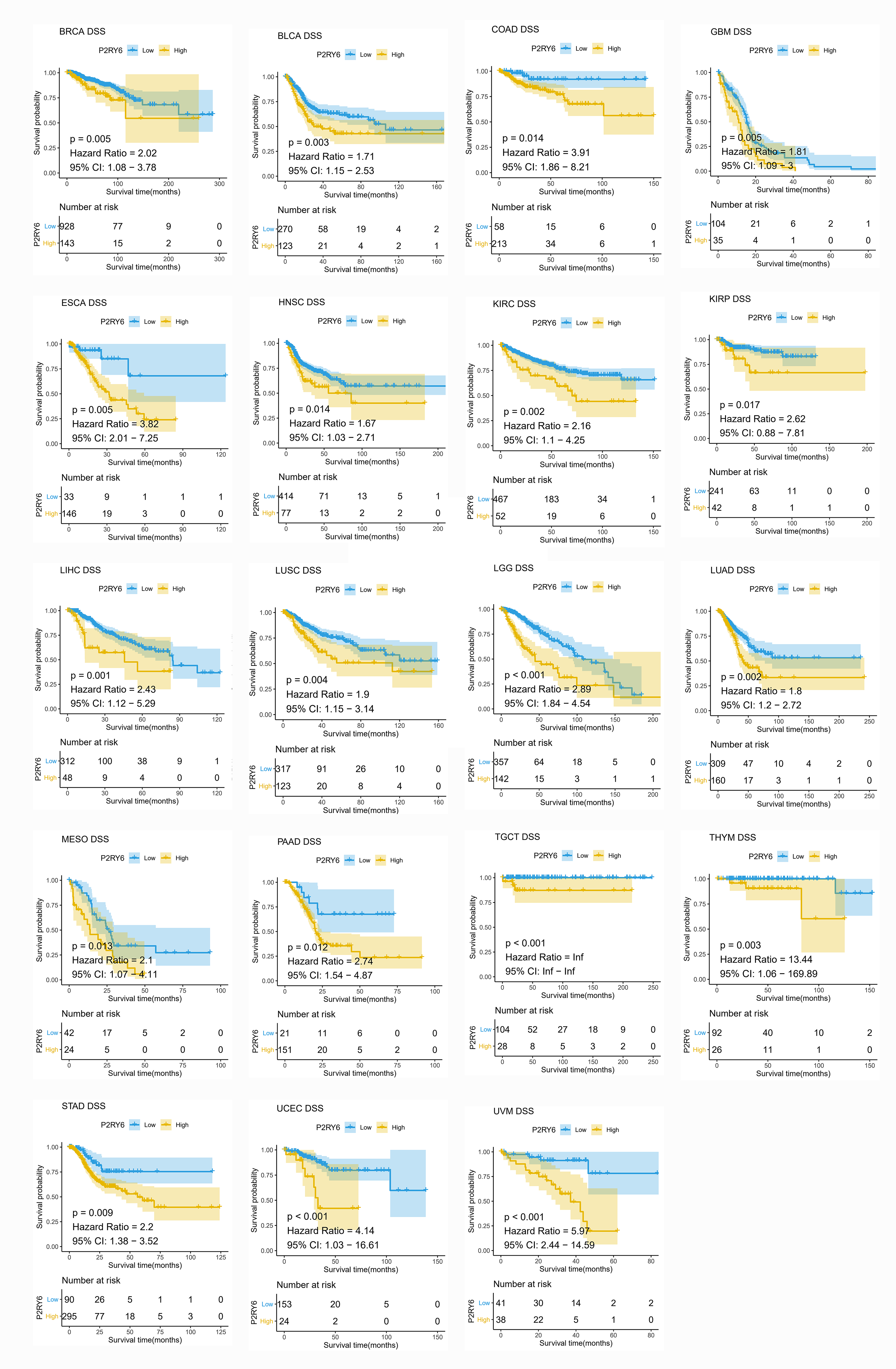

Supplement: Supplementary file 4 — Additional file 4: Supplementary Figure 4. Kaplan-Meier survival curve of DSS in difference of various tumor types. [file 12957_2023_3216_MOESM4_ESM.tif]

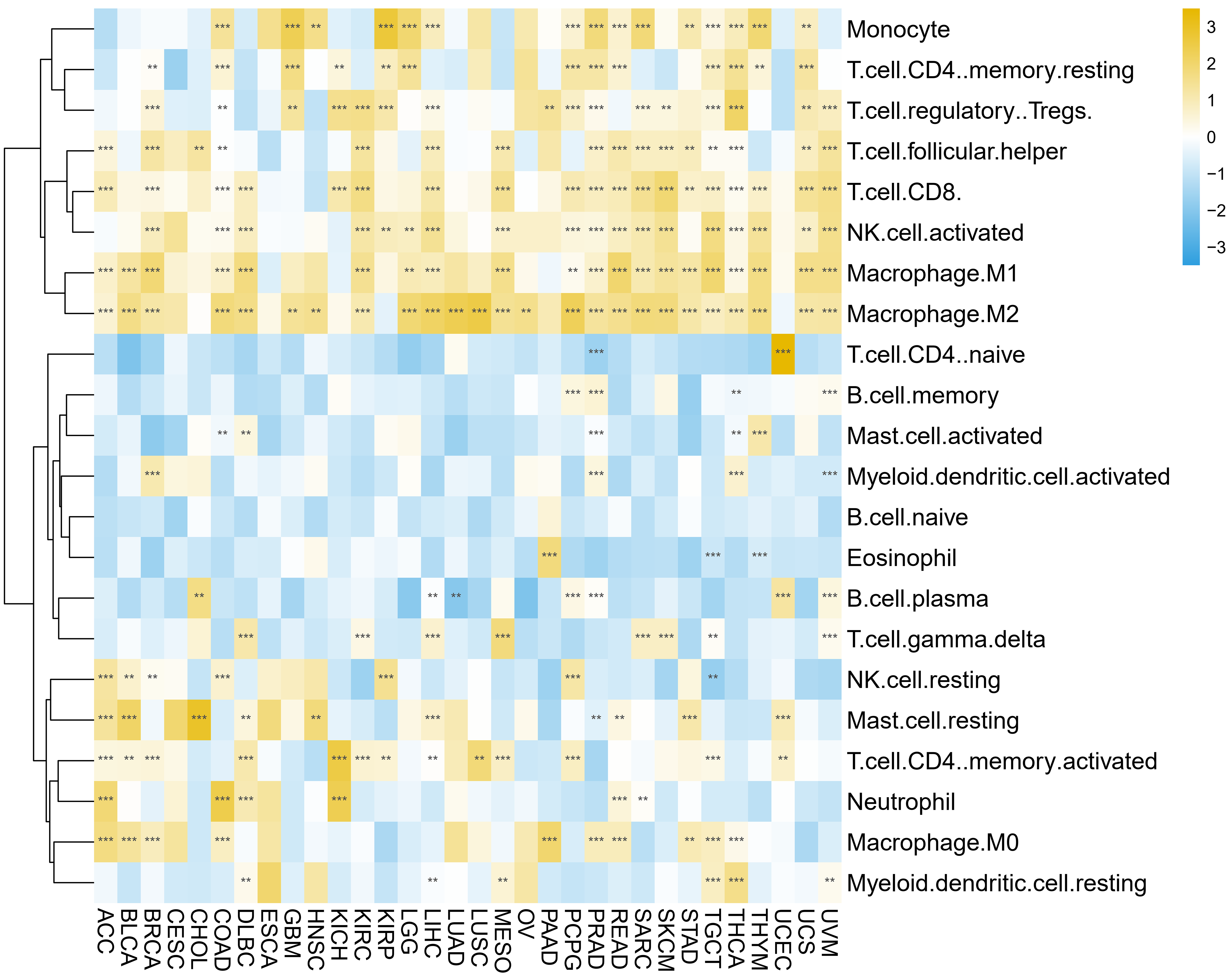

Supplement: Supplementary file 5 — Additional file 5: Supplementary Figure 5. The results of correlation analysis between the expression of P2RY6 in pan-cancer and the impact of immune microenvironment are related to the level of immune cell infiltration. Positive correlation is represented by yellow, and negative correlation is represented by blue, and the darker the color, the stronger the correlation. *, p <0.05, **, p <0.01, and ***, p <0.001. [file 12957_2023_3216_MOESM5_ESM.tif]

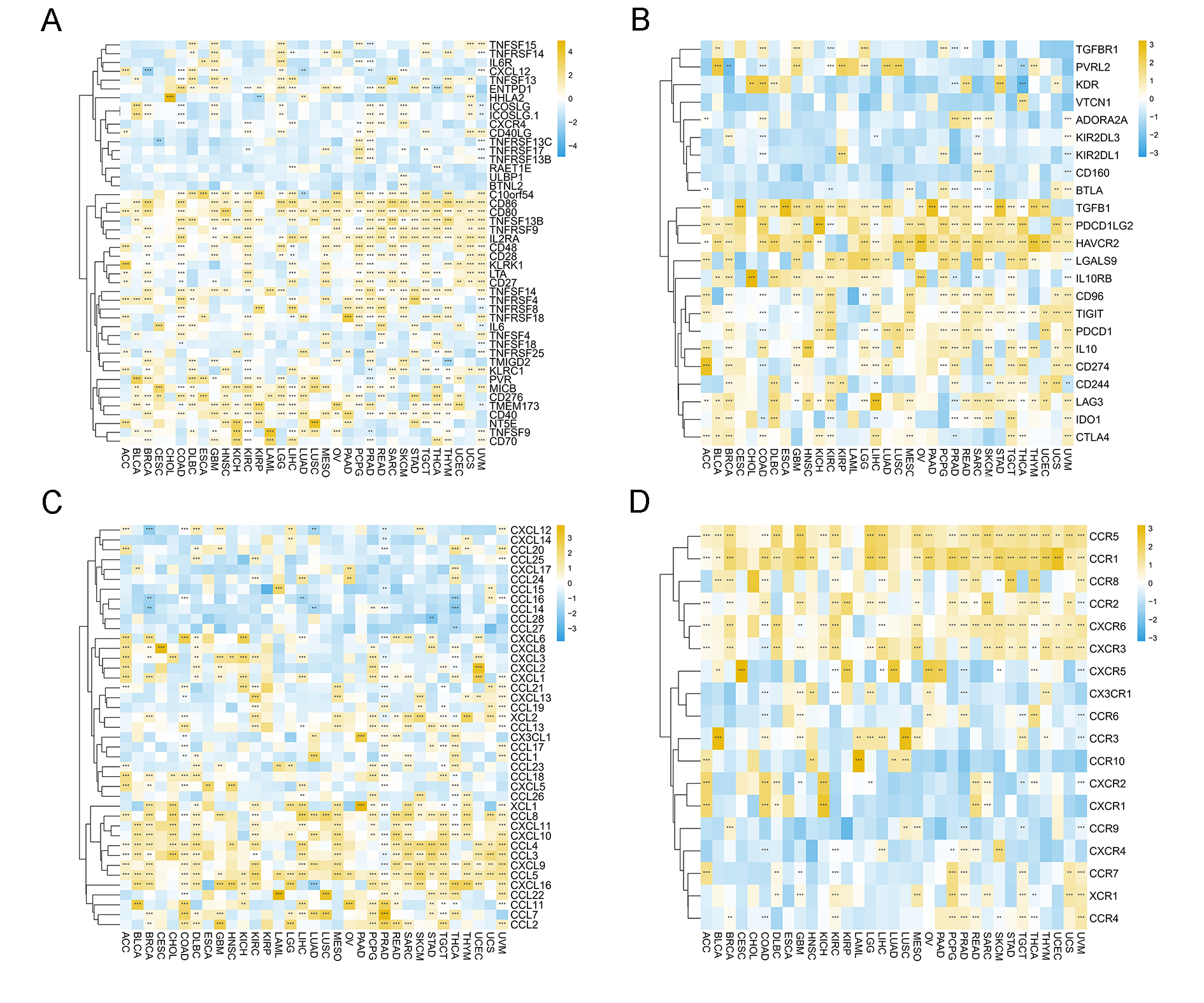

Supplement: Supplementary file 6 — Additional file 6: Supplementary Figure 6. Correlation analysis between P2RY6 expression and immune regulation-related genes. (A) Heat map of the correlation between P2RY6 expression in pan-cancer and immune activation genes. (B) The heat map of the correlation between the expression of P2RY6 in pan-cancer and the immunosuppressive state-related genes. (C) Heat map of the correlation between the expression of P2RY6 in pan-cancer and chemokine genes. (D) Heat map of the correlation between the expression of P2RY6 in pan-carcinoma and chemokine receptor genes. Positive correlation is represented by yellow, and negative correlation is represented by blue, and the darker the color, the stronger the correlation. *, p <0.05, **, p <0.01, and ***, p<0.001. [file 12957_2023_3216_MOESM6_ESM.tif]

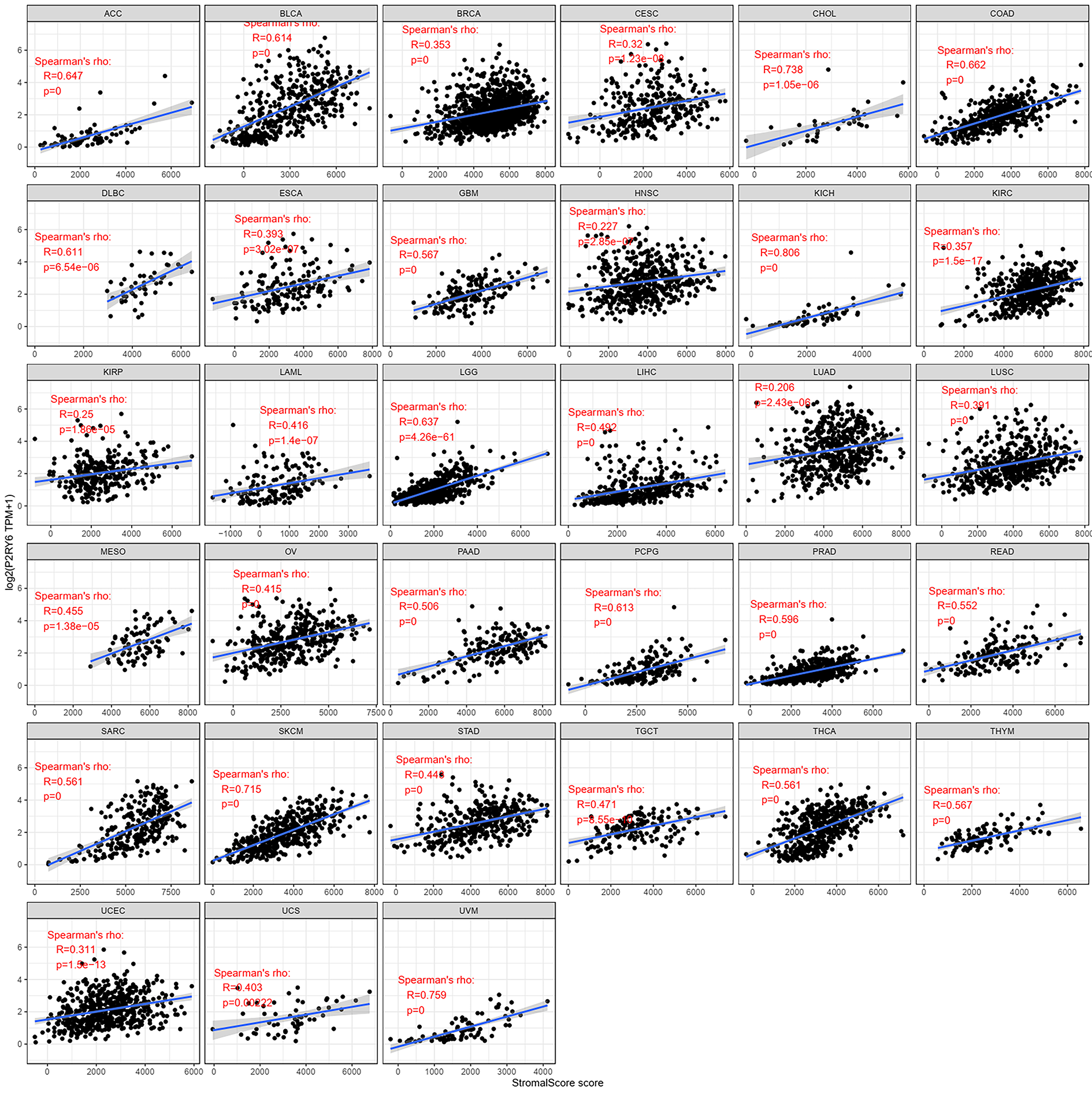

Supplement: Supplementary file 7 — Additional file 7: Supplementary Figure 7. Results of correlation analysis between P2RY6 expression and Stromal Score in pan-cancer. [file 12957_2023_3216_MOESM7_ESM.tif]

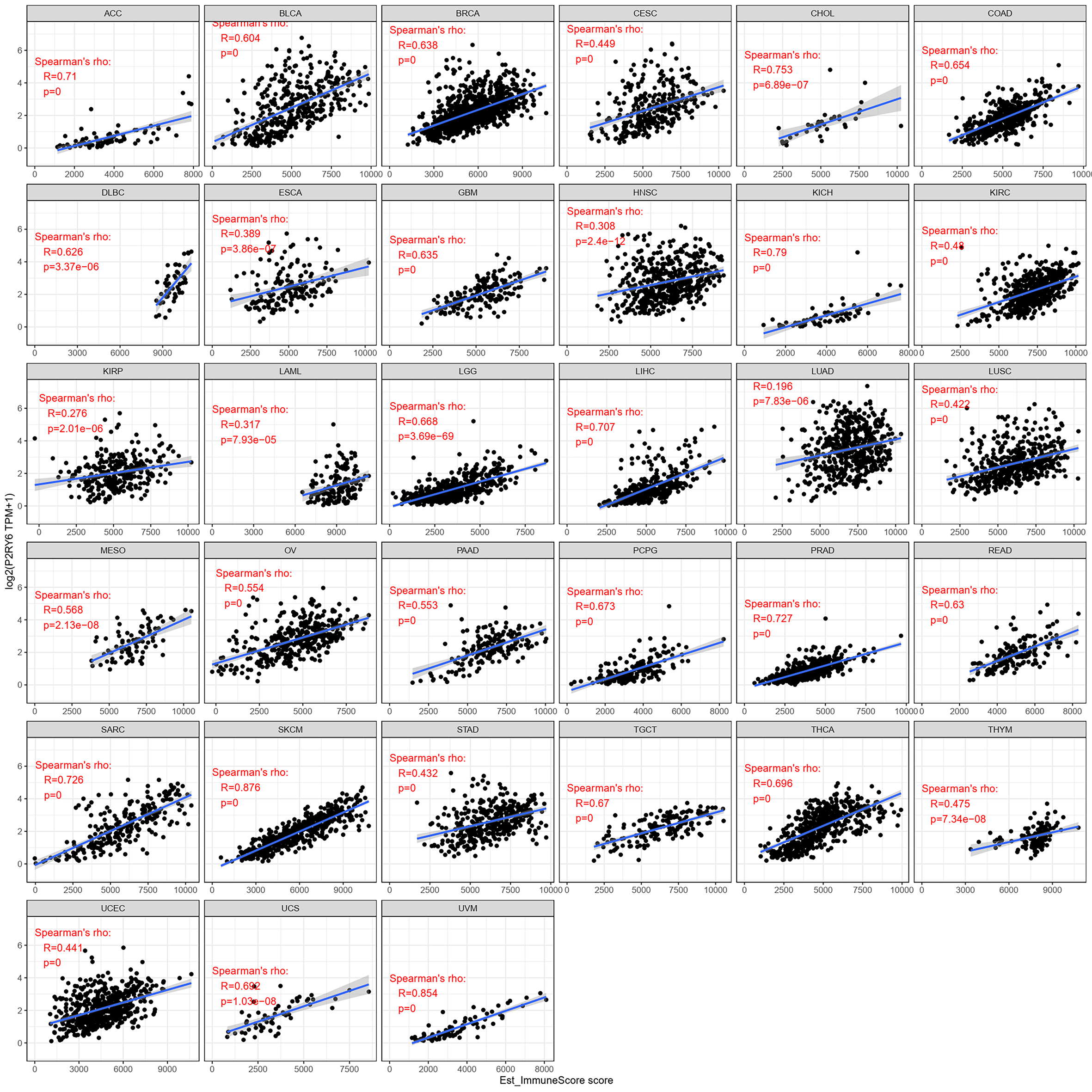

Supplement: Supplementary file 8 — Additional file 8: Supplementary Figure 8. Results of correlation analysis between P2RY6 expression and Immune Score in pan-cancer. [file 12957_2023_3216_MOESM8_ESM.tif]

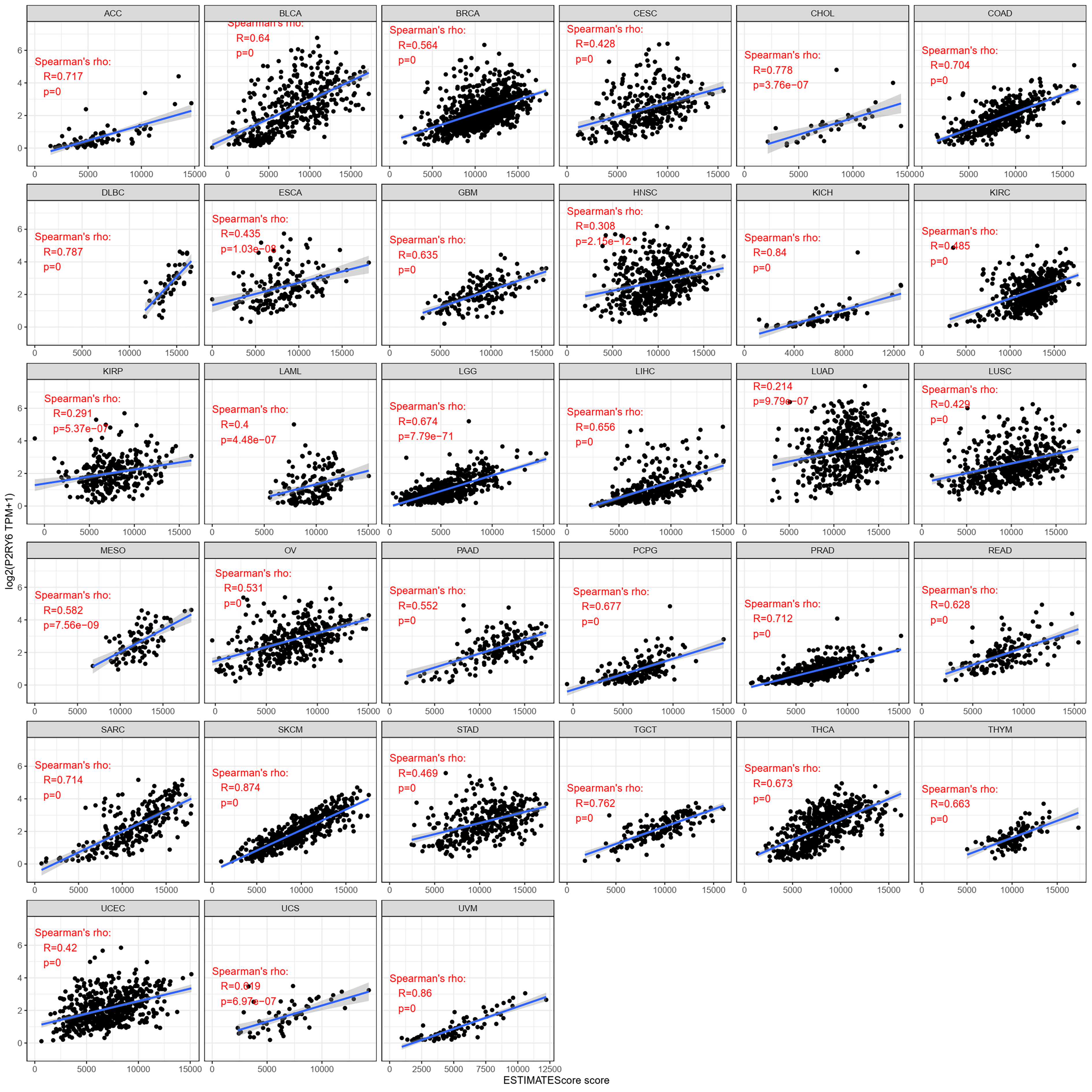

Supplement: Supplementary file 9 — Additional file 9: Supplementary Figure 9. Results of correlation analysis between P2RY6 expression and ESTIMATE Score in pan-cancer. [file 12957_2023_3216_MOESM9_ESM.tif]

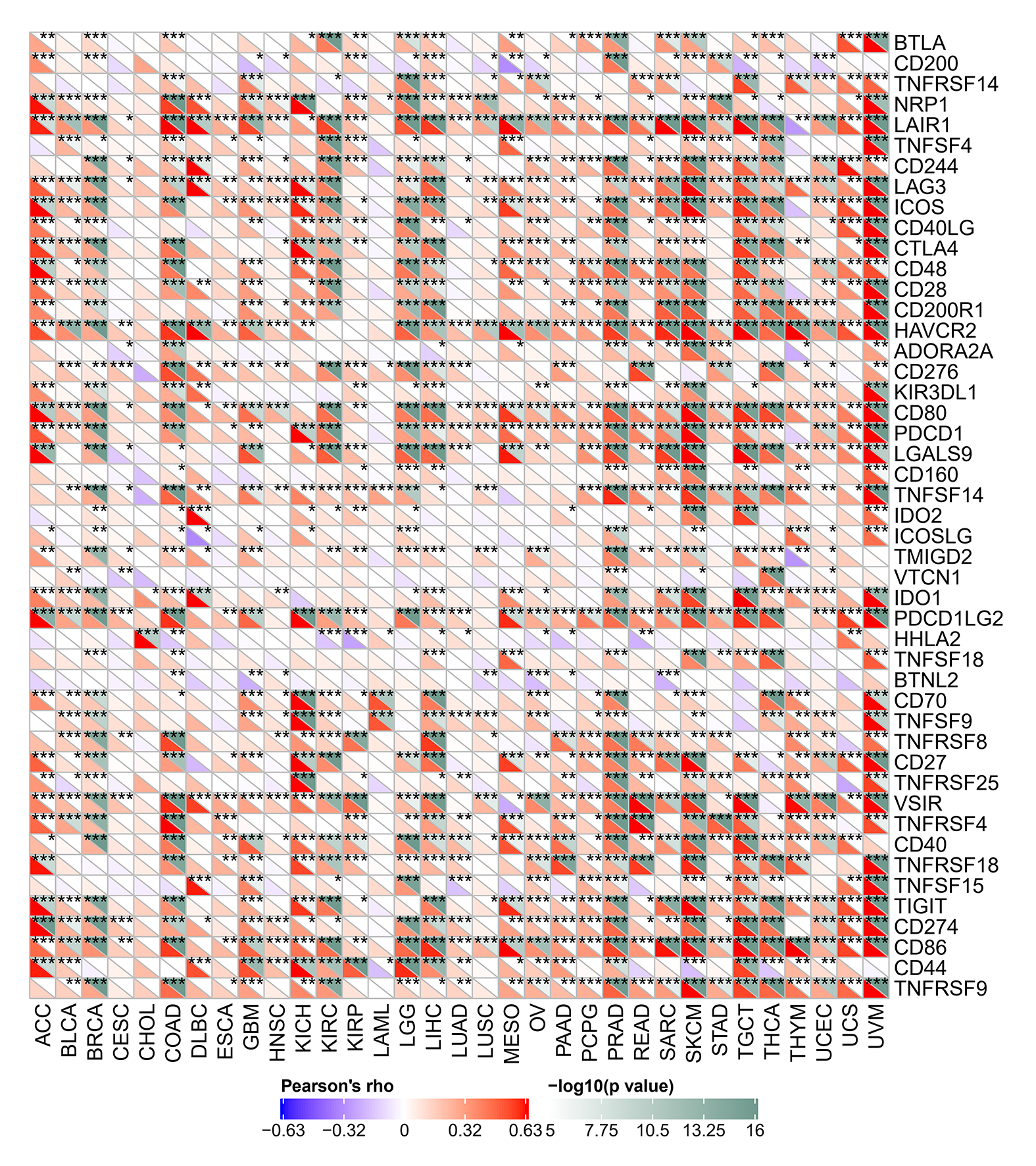

Supplement: Supplementary file 10 — Additional file 10: Supplementary Figure 10. Correlation analysis between P2RY6 expression and pan-cancer immune marker set. *p<0.05; **p <0.01; *** p <0.001. [file 12957_2023_3216_MOESM10_ESM.tif]

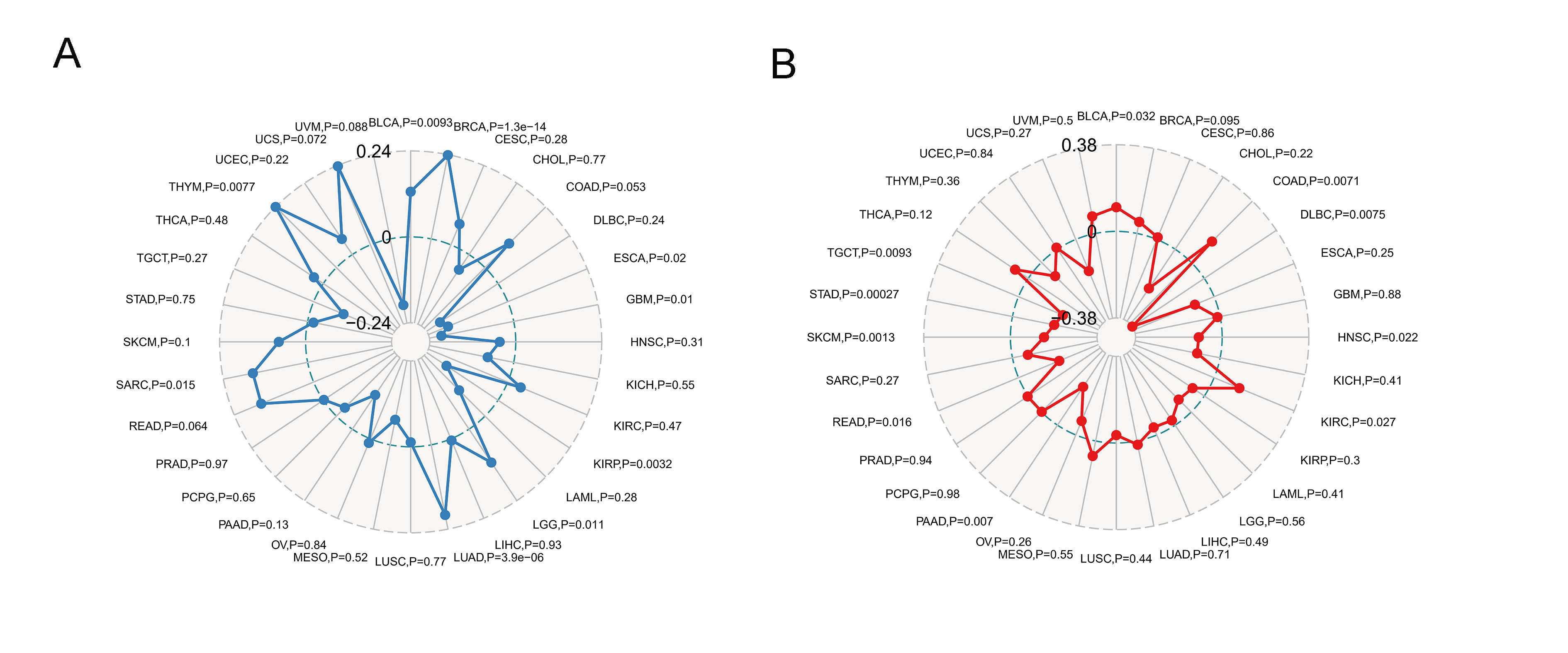

Supplement: Supplementary file 11 — Additional file 11: Supplementary Figure 11. The relationship between P2RY6 expression and TMB and MSI in pan-cancer. (A) TMB. (B) MSI. TMB, tumor mutation burden; MSI, microsatellite instability. The radar chart shows Spearman's correlation coefficient and p-value. [file 12957_2023_3216_MOESM11_ESM.tif]

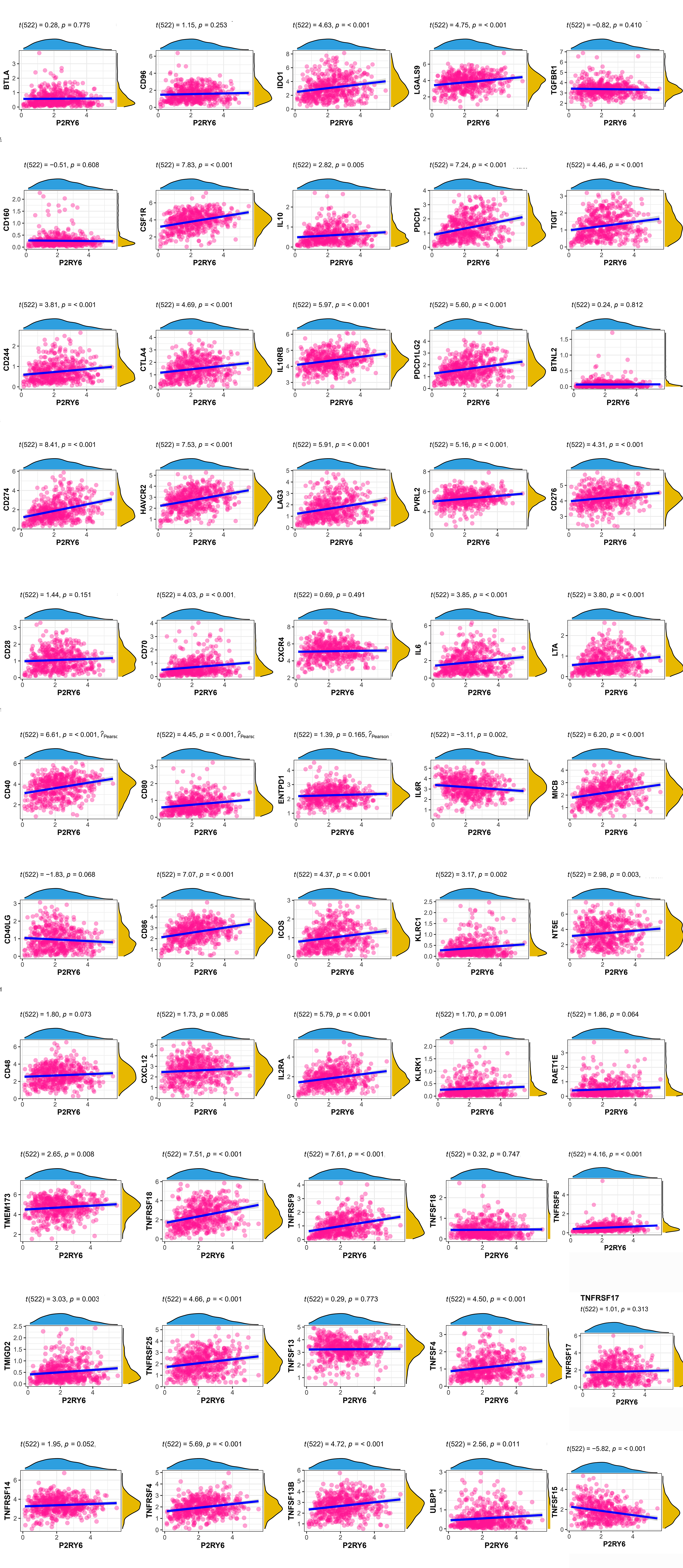

Supplement: Supplementary file 12 — Additional file 12: Supplementary Figure 12. Correlation analysis between P2RY6 gene and immunomodulator. [file 12957_2023_3216_MOESM12_ESM.tif]

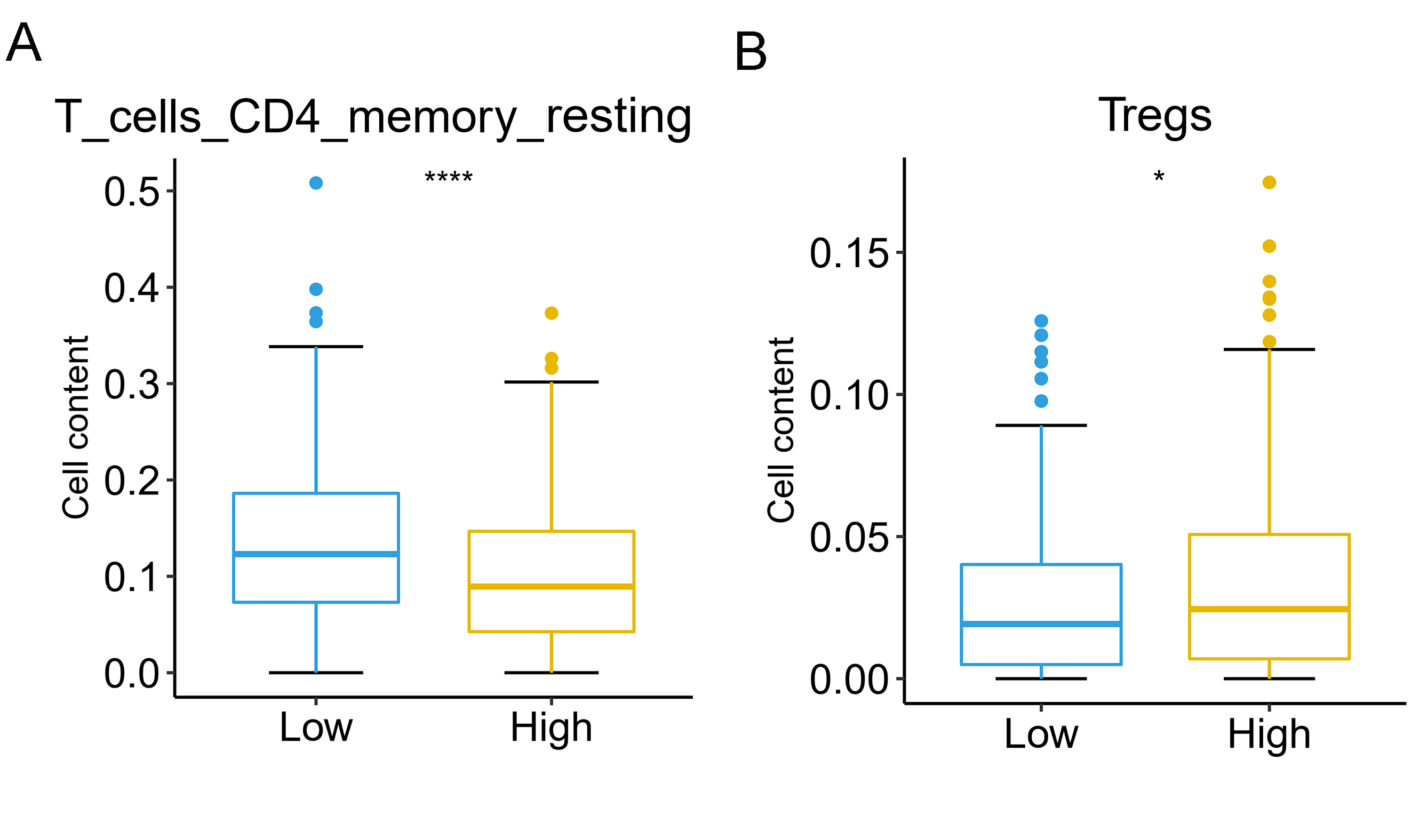

Supplement: Supplementary file 13 — Additional file 13: Supplementary Figure 13. Relationship between P2RY6 expression level and tumor infiltrating immune cells in LUAD. (A) The level of resting T cell infiltration in CD4. (B) The infiltration level of Treg cells. *p <0.05; **p <0.01; *** p <0.001. [file 12957_2023_3216_MOESM13_ESM.tif]

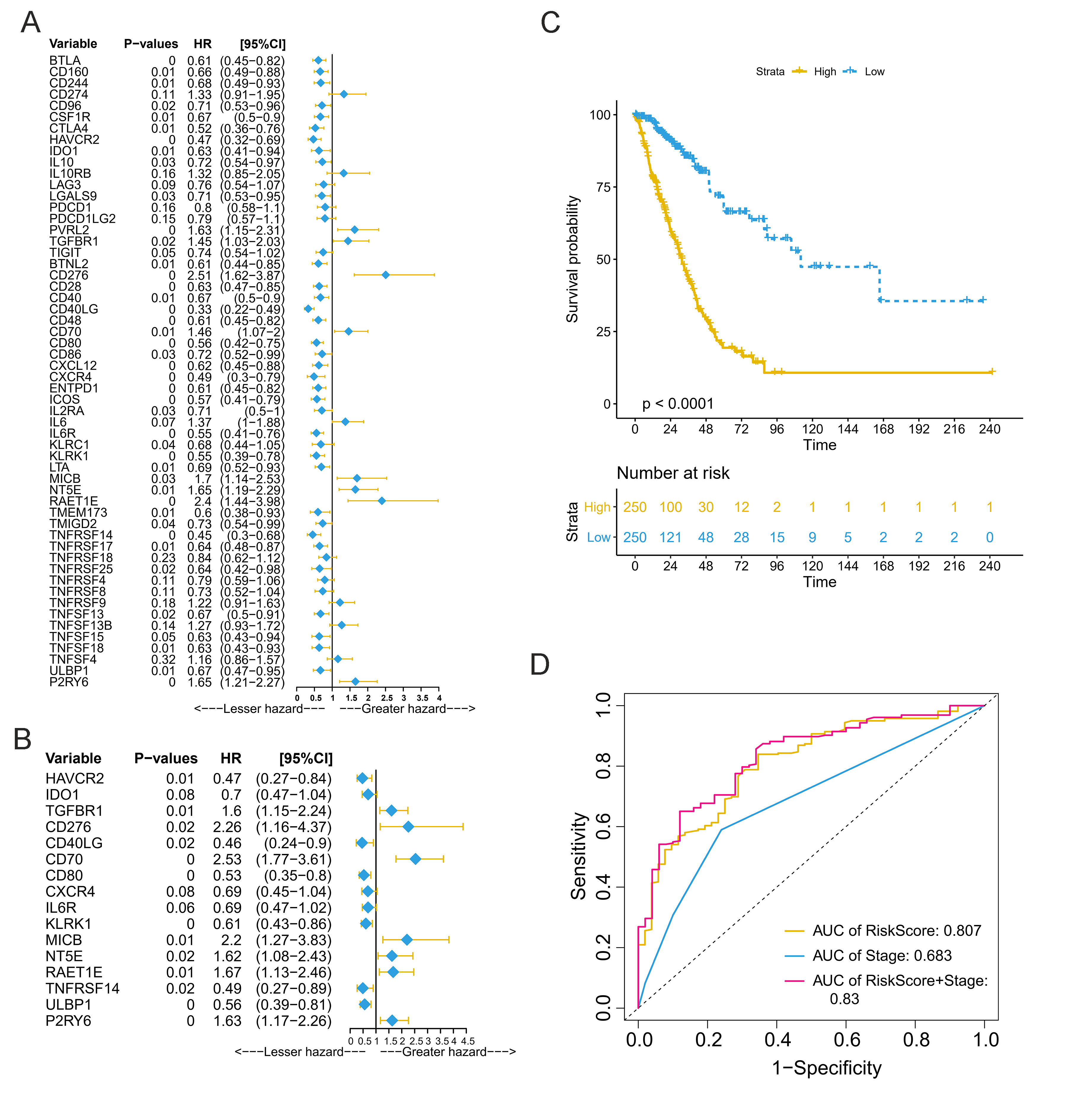

Supplement: Supplementary file 14 — Additional file 14: Supplementary Figure 14. The construction of P2RY6 related risk model. (A) Forest plot results of univariate Cox regression analysis for patients with LUAD. (B) Forest plot results of multivariate Cox regression analysis for patients with LUAD. (C) Kaplan-Meier curve for predicting OS in patients with LUAD based on the TCGA data set. (D) Receiver operating characteristic curve (ROC) analysis results of patients with LUAD. [file 12957_2023_3216_MOESM14_ESM.tif]
